# Characterization of a bacteriophage with broad host range against strains of *Pseudomonas aeruginosa* isolated from domestic animals

**DOI:** 10.1186/s12866-019-1481-z

**Published:** 2019-06-17

**Authors:** Anna Cristhina Carmine de Melo, Amanda da Mata Gomes, Fernando L. Melo, Daniel M. P. Ardisson-Araújo, Agueda Palmira Castagna de Vargas, Valessa Lunkes Ely, Elliot W. Kitajima, Bergmann M. Ribeiro, José Luiz Caldas Wolff

**Affiliations:** 1 CCBS - Curso de Ciências Biológicas, Laboratório de Biologia Molecular e Virologia, Prédio 28, primeiro andar, Universidade Presbiteriana Mackenzie, Rua da Consolação, 896, Consolação, São Paulo, SP CEP 01302-907 Brazil; 2 Departamento de Biologia Celular, Instituto de Ciências Biológicas, Universidade de Brasília, Brasília, DF Brazil; 3 Departamento de Bioquímica e Biologia Molecular, Universidade Federal de Santa Maria, Av. Roraima, 1000, Cidade Universitária, Santa Maria, RS CEP 97105-900 Brazil; 4 Departamento de Medicina Veterinária Preventiva (DMVP), Centro de Ciências Rurais (CCR)Avenida Roraima, Universidade Federal de Santa Maria, 1000. Prédio 44, Sala 5137, Santa Maria, RS CEP 97105-900 Brazil; 5 NAP/MEPA, Departamento de Fitopatologia e Nematologia, ESALQ, Universidade de São Paulo, Piracicaba, SP Brazil

## Abstract

**Background:**

*Pseudomonas aeruginosa* is an opportunistic pathogen and one of the leading causes of nosocomial infections. Moreover, the species can cause severe infections in cystic fibrosis patients, in burnt victims and cause disease in domestic animals. The control of these infections is often difficult due to its vast repertoire of mechanisms for antibiotic resistance. Phage therapy investigation with *P. aeruginosa* bacteriophages has aimed mainly the control of human diseases. In the present work, we have isolated and characterized a new bacteriophage, named Pseudomonas phage BrSP1, and investigated its host range against 36 *P. aeruginosa* strains isolated from diseased animals and against *P. aeruginosa* ATCC strain 27853.

**Results:**

We have isolated a *Pseudomonas aeruginosa* phage from sewage. We named this virus Pseudomonas phage BrSP1. Our electron microscopy analysis showed that phage BrSP1 had a long tail structure found in members of the order Caudovirales. “In vitro” biological assays demonstrated that phage BrSP1 was capable of maintaining the *P. aeruginosa* population at low levels for up to 12 h post-infection. However, bacterial growth resumed afterward and reached levels similar to non-treated samples at 24 h post-infection. Host range analysis showed that 51.4% of the bacterial strains investigated were susceptible to phage BrSP1 and efficiency of plating (EOP) investigation indicated that EOP values in the strains tested varied from 0.02 to 1.72. Analysis of the phage genome revealed that it was a double-stranded DNA virus with 66,189 bp, highly similar to the genomes of members of the genus *Pbunavirus*, a group of viruses also known as PB1-like viruses.

**Conclusion:**

The results of our “in vitro” bioassays and of our host range analysis suggested that Pseudomonas phage BrSP1 could be included in a phage cocktail to treat veterinary infections. Our EOP investigation confirmed that EOP values differ considerably among different bacterial strains. Comparisons of complete genome sequences indicated that phage BrSP1 is a novel species of the genus *Pbunavirus*. The complete genome of phage BrSP1 provides additional data that may help the broader understanding of pbunaviruses genome evolution.

**Electronic supplementary material:**

The online version of this article (10.1186/s12866-019-1481-z) contains supplementary material, which is available to authorized users.

## Background

*Pseudomonas aeruginosa* is a Gram-negative free-living and parasitic bacterium widespread in the environment. The species is an opportunistic pathogen of great concern to human health due to its broad spectrum of antibiotic resistance and diverse virulence factors. *P. aeruginosa* is the primary cause of respiratory infection in cystic fibrosis patients and one of the most important causes of severe infections in burn victims [1–3]. Moreover, it is a frequent etiological agent of hospital-acquired diseases, such as pneumonia, urinary tract and surgical site infections [4, 5].

The versatile metabolic capacity of *P. aeruginosa* allows it to multiply in a variety of environments and hosts. A study published in 1952 showed a high occurrence in sewage water samples (90.4%) and a much lower incidence in human feces (11%) and soil samples (3%) [6]. Subsequent investigations confirmed the presence of *P. aeruginosa* in soil [7, 8], in human feces [9] and water [10, 11]. One of the consequences of the presence of *P. aeruginosa* in soil and water is that plants are often in contact with these bacteria. In fact, several plant species can be a source of infection with *P. aeruginosa* [12].

*P. aeruginosa* is also able to infect distantly related species of animals, such as insects [13], nematodes [14], fishes [15] and birds [16]. Moreover, a variety of domestic animals are susceptible to its infection. For instance, *P. aeruginosa* may cause endometritis in mares [17, 18], mastitis in dairy animals [19–21], ear infection, pneumonia, septicemia and enteritis in chinchilla [22] and hemorrhagic pneumonia in confined mink [23–25]. In addition, the species is associated with ear, urinary and skin infection in dogs [26–32]. These infections are often difficult to heal due to multi-antibiotic resistance mechanisms, including the formation of biofilm [33, 34].

The difficulties related to the antibiotic treatment of *P. aeruginosa* and the often damaging nature of its infection have prompted investigations of viruses (bacteriophages) as antimicrobial agents against this species [35, 36]. Of prime interest is the possibility of using bacteriophages to treat *P. aeruginosa* infections in cystic fibrosis patients and burn victims [37, 38]. On the other hand, there are much fewer investigations aiming for veterinary applications. One of these studies used antibiotic-resistant strains isolated from dogs to screen phages with potential therapeutic value [39]. Another investigation showed evidence of successful application of bacteriophages in the treatment of canine otitis [40].

Here we present our work with a new *P. aeruginosa* bacteriophage, named Pseudomonas phage BrSP1. We studied the infectivity and host range of phage BrSP1 against *P. aeruginosa* strains isolated from domestic animals (including dogs, cattle, swine, horse, and chinchilla) and *P. aeruginosa* ATCC strain 27853. Our experiments demonstrated that this phage was able to infect 51.4% of the bacterial strains analyzed. The EOP values for phage BrSP1 varied greatly, reaching approximately one hundred fold difference between the strains with the highest and lowest EOPs. In the conditions used in our “in vitro” bioassays, infection with phage BrSP1 maintained low levels of the bacterial population for at least 12 h post-infection. Sequence analysis indicated that phage BrSP1 is a putative new virus species with 66,189 bp, belonging to the genus *Pbunavirus* of the family *Myoviridae*. Members of this genus are also referred to as PB1-like viruses [41, 42]. Our results indicated that BrSP1 could be part of a phage cocktail to control *P. aeruginosa* infection in domestic animals. Moreover, the complete genome of phage BrSP1 provided additional data that may help the broader understanding of pbunavirus genome.

## Results

### Phage isolation, morphology and storage stability

**Fig. 1:**
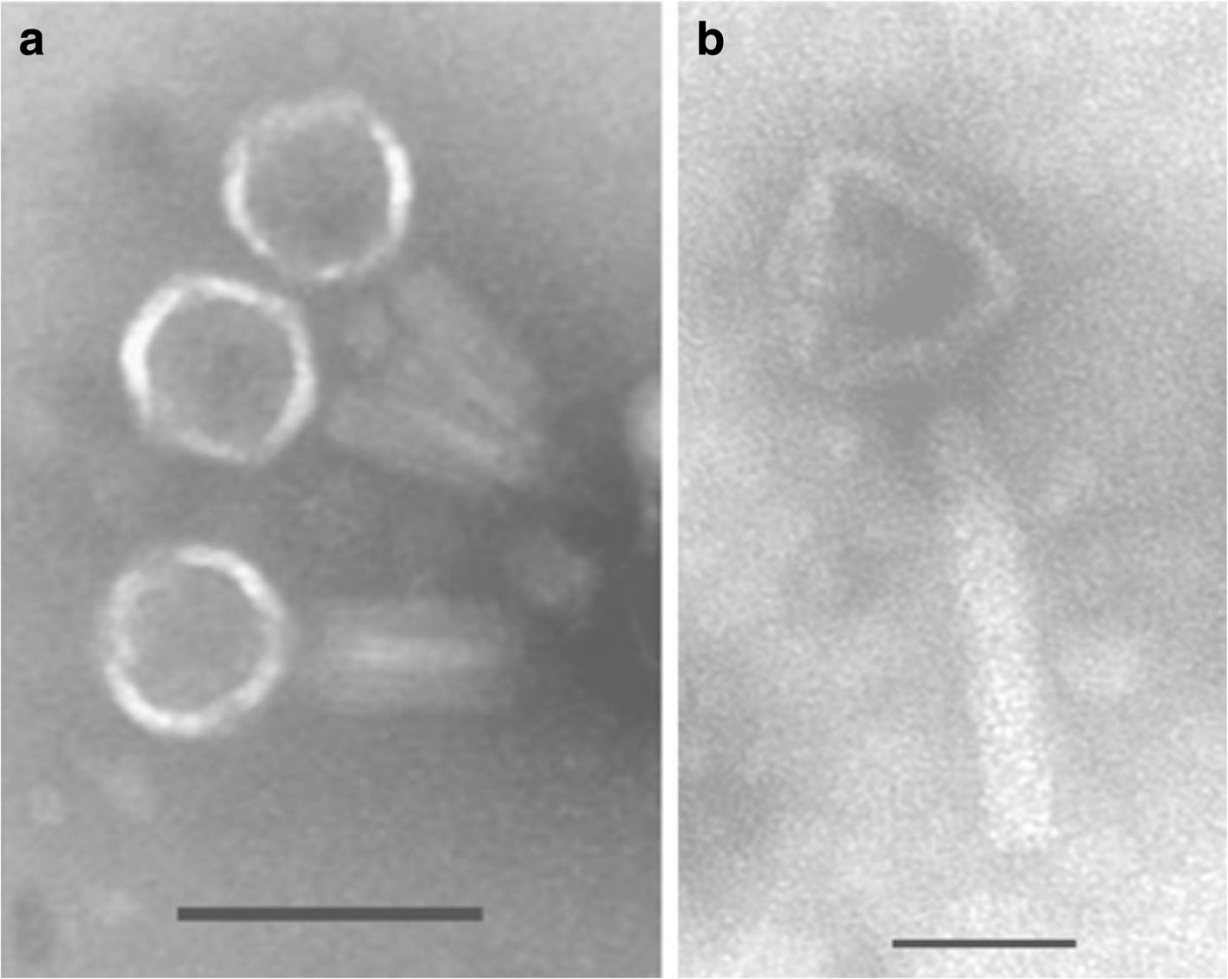
Electron microscopy of phage BrSP1 negatively stained with uranyl acetate. The scale bar represents 100 nm (**a**) and 50 nm (**b**)

One lytic phage against *P. aeruginosa* strain Lfar01 was isolated from sewage and a homogeneous stock prepared after three rounds of purification. We named this virus Pseudomonas phage BrSP1. Electron microscopy of phage BrSP1 showed the long tail structure typical of members of the order Caudovirales (Fig. 1). The titer of a Phage BrSP1 stock had a 12.4 fold reduction upon storage, under refrigerated temperature (approximately 5 °C), for 104 days (Additional file 1: Table S1).

### Host range investigation and EOP analysis

We evaluated host range using, at first, spot test with undiluted phage stock and 37 *P. aeruginosa* strains, which included 36 strains isolated from domestic animals and *P. aeruginosa* ATCC 27853 (Table 1 and Additional file 2: Table S2). This preliminary analysis resulted in clear or turbid zones in 24 of the tested strains (Table 1). Subsequently, we inoculated these 24 strains with doses of diluted phage. These later experiments indicated that five of the samples were, in fact, resistant to phage BrSP1 (Table 1). The clear zones produced initially in these five strains were likely due to the disruption of cell membranes [43] or to bacteriocins produced during phage stock production. The formation of individual lysis plaques, due to the dilution of the phage stock, confirmed the susceptibility of 19 strains (51.4%), including *P. aeruginosa* ATCC 27853 (Table 1). These results, therefore, confirmed the importance of using low titer viral stocks when investigating host range, as has been previously noted [44].

**Table 1:** Investigation of the susceptibility to phage BrSP1

Isolate ^1^	Undiluted phage ^2^	Diluted phage ^3^
SWSM01	Not clear	–
SWSM02	Not clear	–
SWSM03	Not clear	–
SWSM04	Not clear	–
ATCC 27853	Clear	Plaques
CAPE01	Clear	Plaques
CAPE02	Clear	Plaques
BOIJ01	Clear	Plaques
BOIJ02	Clear	Plaques
CASM01	Not clear	–
CASM02	Turbid	–
CASM03	Turbid	Plaques
CASM04	Not clear	–
CASM05	Clear	Plaques
CASM06	Clear	Plaques
BOJC01	Clear	Plaques
EQSM01	Clear	Plaques
EQSM02	Clear	Plaques
CASM07	Clear	Plaques
BOSV01	Turbid	–
BOIJ03	Clear	Plaques
BOCA01	Clear	Plaques
CASM08	Not clear	–
CASM09	Not clear	–
BOPS01	Clear	–
BOSJ01	Clear	Plaques
BOCP01	Not clear	–
BOCP02	Not clear	–
CASM10	Clear	Plaques
BOSM01	Turbid	Plaques
ROSM01	Not clear	–
CASM11	Turbid	Plaques
CASM12	Not clear	–
SWSM05	Not clear	–
BOSJ02	Clear	Plaques
CASP01	Clear	–
CASM13	Turbid	–
Lfar01 ^4^	Clear	Plaques

^1^Additional file 2: Table S2 shows the origins of the isolates

^2^Spot test done with undiluted phage stock (approximately 4.5 × 10^5^ PFU). Results were evaluated as “Not clear” when no inhibition zone was observed; “Clear” when complete clearing was observed; “Turbid” when the inhibition zone was mostly turbid

^3^Spot test done with diluted phage stock (up to 10^6^ dilution). The formation of individual lysis plaques is indicated

^4^Strain Lfar01 was used as a control

**Fig. 2:**
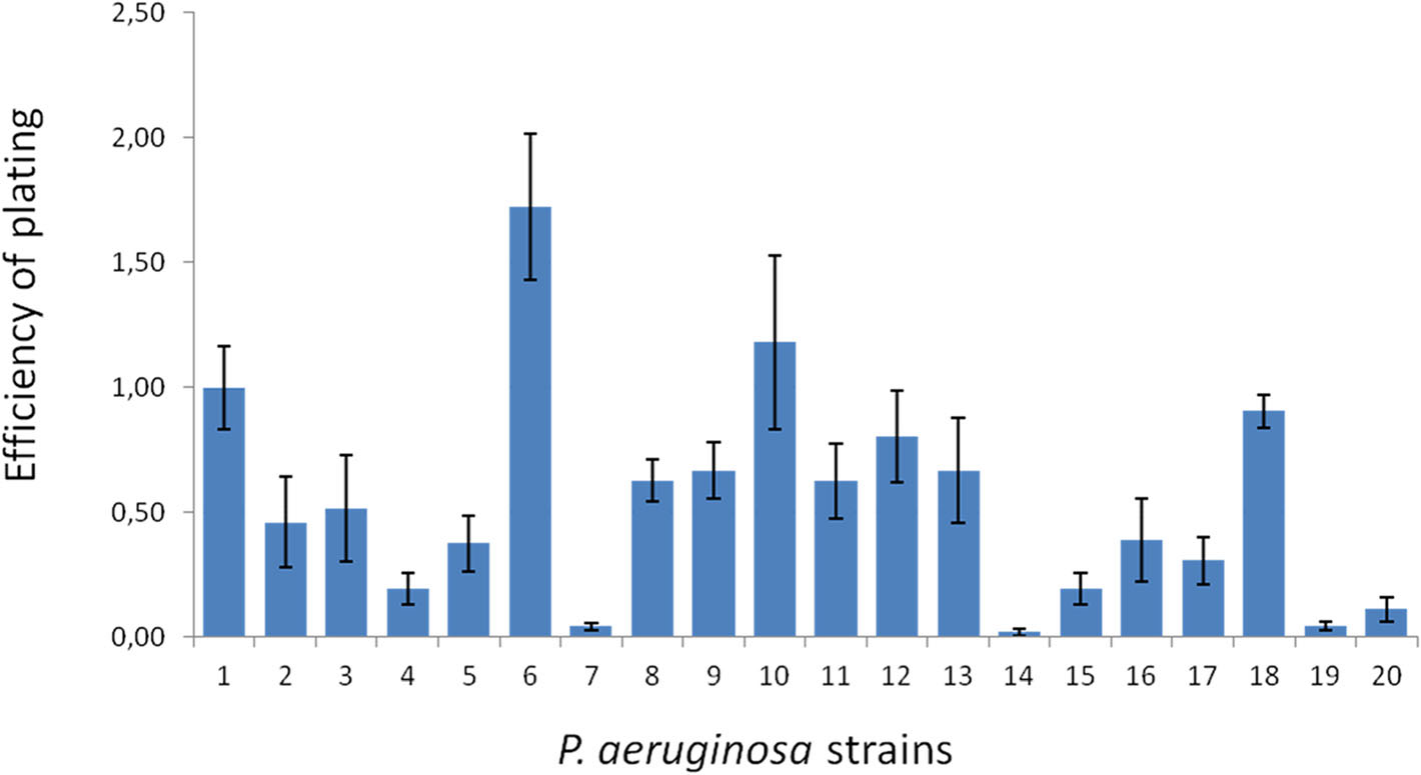
Efficiency of plating relative to the number of plaques produced in *P. aeruginosa* strain ATCC 27853. The EOP was evaluated for 20 strains of *P. aeruginosa*. The numbers on the vertical lines indicate EOP values and the numbers on the horizontal line indicate the *P. aeruginosa* strain. Sample 1 is the reference sample (*P. aeruginosa* strain ATCC 27853). The other samples are: 2: Lfar01, 3: CAPE01, 4: CAPE02, 5: BOIJ01, 6: BOIJ02, 7: CASM03, 8: CASM05, 9: CASM06, 10: BOJC01, 11: EQSM01, 12: EQSM02, 13: CASM07, 14: BOIJ03, 15: BOCA01, 16: BOSJ01, 17: CASM10, 18: BOSM01 19: CASM11, 20: BOSJ02. The dilution 10^4^ of the stock of phage BrSP1 was used in the assays, except for samples 7, 14 and 19, for which a 10 times more concentrated viral dose was used. The results presented are the average of three assays. Error bars correspond to the standard deviation

We calculated EOP values as the ratio of lysis plaques produced in each susceptible strain (for a fixed dose of phages) divided by the number of plaques produced in *P. aeruginosa* strain ATCC 27853 (Fig. 2). In these assays, we inoculated most of the samples with ten microliters of dilution 10^4^ of our phage stock. As this dose did not produce plaques on strains CASM03, BOIJ03 and CASM11, we used a dose that was ten times higher (dilution 10^3^) in their analysis.

### Molecular characterization of bacterial strains

The 16S rRNA gene of the strains that were not susceptible to phage BrSP1 infection were PCR amplified and sequenced. Blast analysis of the sequences confirmed the resistant strains were indeed *P. aeruginosa* (data not shown). The Genbank accession numbers of these sequences are available in Additional file 3: Table S3.

### “In vitro” lytic activity of phage BrSP1 and origin of the bacteriophage resistant mutants

**Fig. 3:**
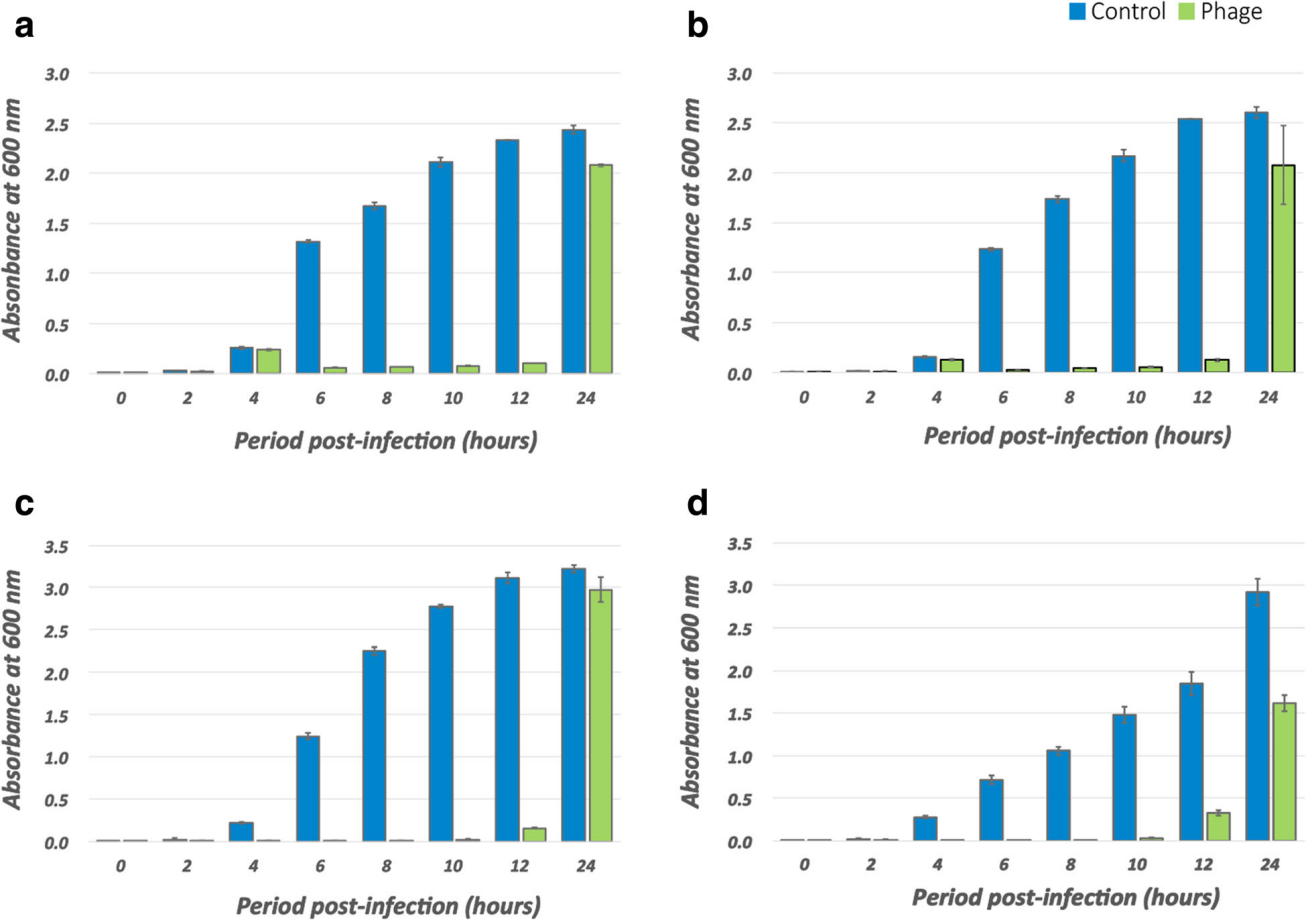
Effect of bacteriophage BrSP1 in the multiplication of *P. aeruginosa* strains Lfar01, ATCC 27853 and BOIJ02 over a 24 h period. The multiplication of *P. aeruginosa* strains in broth cultures was evaluated in the presence and in the absence of bacteriophage BrSP1. **a**: Assay done with *P. aeruginosa* strain Lfar01 and a viral dose of 1 million PFU per mL; **b**: Assay done *P. aeruginosa* strain Lfar01 and a viral dose with 10 million PFU per mL; **c**: Assay done with *P. aeruginosa* strain ATCC 27853 and a viral dose of 10 million PFU per mL; **d**: Assay done with *P. aeruginosa* strain BOIJ02 and a viral dose of 10 million PFU per mL. The results presented in the figure are the mean of three assays. Error bars correspond to the standard deviation

We investigated the lytic activity of phage BrSP1 in assays with *P. aeruginosa* strains Lfar01, ATCC 27853 and BOIJ02, a strain with high EOP. In these assays, there was a remarkable reduction in bacterial cell population and bacterial growth remained checked until approximately 12 h p.i. (Fig. 3). At 24 h p.i., however, the cell density of infected and non-infected samples was almost the same. The tenfold difference in the viral concentration used in some of the assays did not produce any noticeable variation (Fig. 3).

In order to understand the nature of the emergence of resistance observed in our assays, we investigated the frequency of occurrence of bacteriophage insensitive mutants (BIMs) in three *P. aeruginosa* strains. Our results indicated that BIMs arose independently of phage infection at frequencies that ranged from 7.07 × 10^− 9^ (minimum frequency for strain BOIJ02) to 2.10 × 10^− 7^ (maximum frequency for strain Lfar01; Table 2).

**Table 2:** Frequency of the occurrence of spontaneous phage resistant mutants ^1^

*P. aeruginosa* strain	Minimum frequency	Maximum frequency
Lfar01	5.43 × 10^−8^	2.10 × 10^− 7^
ATCC 27853	6.45 × 10^− 9^	1.54 × 10^− 8^
BOIJ02	7.07 × 10^− 9^	1.79 × 10^− 8^

^1^Based on the analysis of five individual samples for each strain

### BrSP1 genome size, GC content and accession number

The sequencing of the Pseudomonas phage BrSP1 genome showed that it was 66,189 bp in length with a G + C content of 55.7%. The Genbank accession number of the annotated genome of phage BrSP1 is MF623055.

### Gene content analysis and genome features

We identified 94 open reading frames (ORFs; Additional file 4: Table S4) in the genome of phage BrSP1. These ORFs were assigned based mainly on previously annotated genomes of closely related phages. We also checked ORFs with greater than 100 nucleotides that initiated with CTG, TTG, and GTG and identified eight putative genes using this criterion (Additional file 4: Table S4). Blast analysis showed that two of the hypothetical genes having alternative start codons (ORFs 48 and 56) had 100% similarities to putative proteins of closely related pbunaviruses (data not shown). The annotation of the genome of phage BrSP1 showed the typical block organization, with groups of genes in the same orientation (Additional file 4: Table S4). Other members of the genus *Pbunavirus* display similar genomic organization [37, 41].

We analyzed codon usage based on the relative synonymous codon usage (RSCU), as described by Sharp and Li [45]. RSCU is the ratio of the actual number of times a codon occurs by the number of times that codon was expected to be used supposing an equal distribution of all codons for that particular amino acid [45]. RSCU values higher than one indicate a codon is used more frequently than expected (positive bias), whereas values smaller than one indicate a codon that is used less frequently than expected (negative bias). Our analysis showed that phage BrSP1 codon preferences were, in general, similar to those of the host (Additional file 5: Table S5). Overt differences were observed in few cases as, for instance, with the TAA stop codon and the ACT codon for threonine. Both of these codons display a strong negative bias in *P. aeruginosa* [46], but showed positive bias (TAA) or no bias (ACT) in phage BrSP1 (Additional file 5: Table S5).

**Fig. 4:**
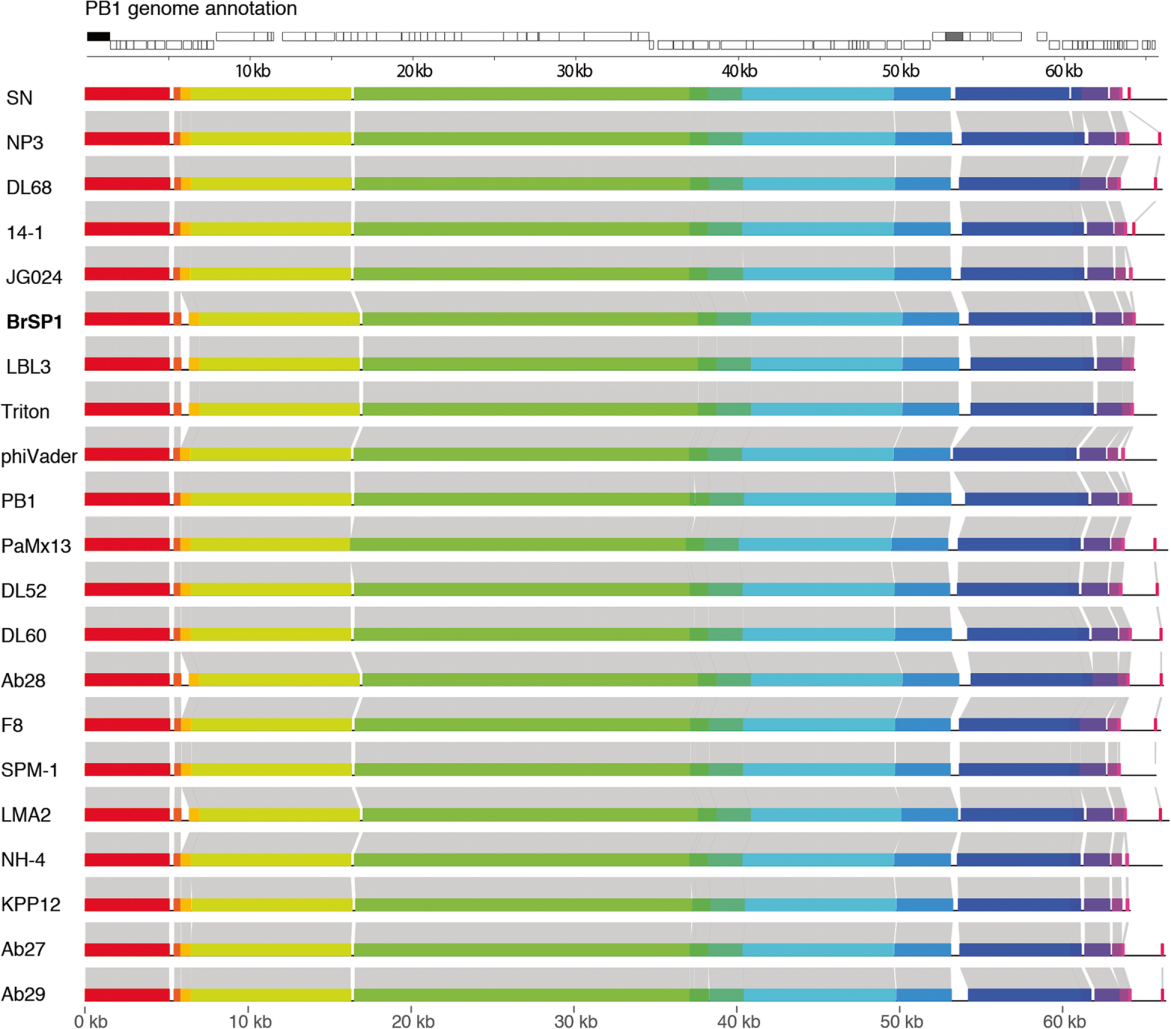
Whole genome synteny analysis of pbunaviruses genomes. Schematic genome alignment diagram obtained using the Mauve software package and plotted using genoPlotR. The analysis was performed using 20 complete genomes available in Genbank, as described in Table 3, and with the phage BrSP1 genome. To facilitate synteny analysis, the genomes were oriented to start at the *terminase* gene (highlighted in black) to overcome the uncertainties in the genome start of several isolates. Gray shading indicates homologous regions among genomes, colored bars represent nucleotide syntenic blocks, and the white spaces between blocks indicate missing regions. All viruses were collinear. The PB1-ORF070 is annotated and highlighted in black

We performed a whole genome comparison among all genomes selected for phylogenetic analysis using the MAUVE method (Fig. 4). In this analysis, the genomes were oriented to start at the terminase gene to overcome the uncertainties in the genome start of several isolates. The genomic architecture of all viruses showed a conserved collinear region flanked by less conserved areas towards the extremities (Fig. 4). All viruses analyzed here showed limited genomic diversity, but some long indels (> 1 kb) were observed, such as the indel resulting in the loss or gain of PB1-ORF070 (depending on the genome), which codes for a hypothetical protein related to chaperone DnaJ (Fig. 4, highlighted in black). Overall, the genome of Pseudomonas phage BrSP1 was similar in gene content and length to all members of the genus *Pbunavirus*, suggesting that horizontal gene transfer (HGT) events from other phages and host genomes are not frequent in the members of this genus. This observation is compatible with the low gene flux (LGCF) evolutionary mode proposed by Mavrich and Hatfull [47] for the evolution of lytic phages.

### Classification and phylogeny of phage BrSP1 based on the analysis of the whole genome and of the terminase gene

The International Committee on Taxonomy of Viruses (ICTV) has chosen 95% whole genome DNA sequence identity as the criterion for demarcation of phage species based on the BLASTN algorithm [48]. Whole genome analysis using the Basic Local Alignment Search Tool (BLAST) [49] showed that the phage BrSP1 BLAST best hit was Pseudomonas phage NH-4 (Genbank accession number: JN254800) with the highest percent identity of 97% (coverage of 96%, data not show). In order to classify phage BrSP1 accurately, pairwise genome alignments were performed between all members of *Pbunavirus* genus using PASC [50]. As shown in Table 3, the pairwise analysis yielded lower identity values than those obtained with BLAST, as the percentage identity in the later was based on partial coverage. Pairwise genomic alignment revealed that BrSP1 phage whole genome identity varied from 86% (compared to Pseudomonas phage PaMx13, Table 3) to 94% (compared to Pseudomonas phage JG024, Table 3). As the genome of BrSP1 differed from all the other species by more than the 5% limit proposed by the ICTV phage BrSP1 is a putative novel species of the genus.

**Table 3:** Pairwise comparisons of the genome of Pseudomonas phage BrSP1 against all complete genomes classified as members of *Pbunavirus* genus

Accession number	Virus name	Identity (%)
GU815091	Pseudomonas phage JG024	94.1
FM897211	Pseudomonas phage 14-1	93.4
KR054033	Pseudomonas phage DL68	93.23
FM887021	Pseudomonas phage SN	93.23
JN254800	Pseudomonas phage NH-4	93.2
LN610579	Pseudomonas phage vB_PaeM_PAO1_Ab27	92.99
FM201282	Pseudomonas phage LMA2 complete genome	92.57
AB560486	Pseudomonas phage KPP12 DNA	92.56
LN610588	Pseudomonas phage vB_PaeM_PAO1_Ab29	92.52
KU198331	Pseudomonas phage NP3	91.65
FM201281	Pseudomonas phage LBL3 complete genome	90.66
DQ163917	Pseudomonas phage F8	87.47
KF981875	Pseudomonas phage SPM-1	87.41
KR054030	Pseudomonas phage DL60	86.57
LN610589	Pseudomonas phage vB_PaeM_C1-14_Ab28	86.57
KT254130	Pseudomonas phage phiVader	86.45
JQ067083	Pseudomonas phage PaMx13	86.44
KT372698	Pseudomonas phage Triton	86.43
EU716414	Pseudomonas phage PB1	86.42
KR054028	Pseudomonas phage DL52	86.42

**Fig. 5:**
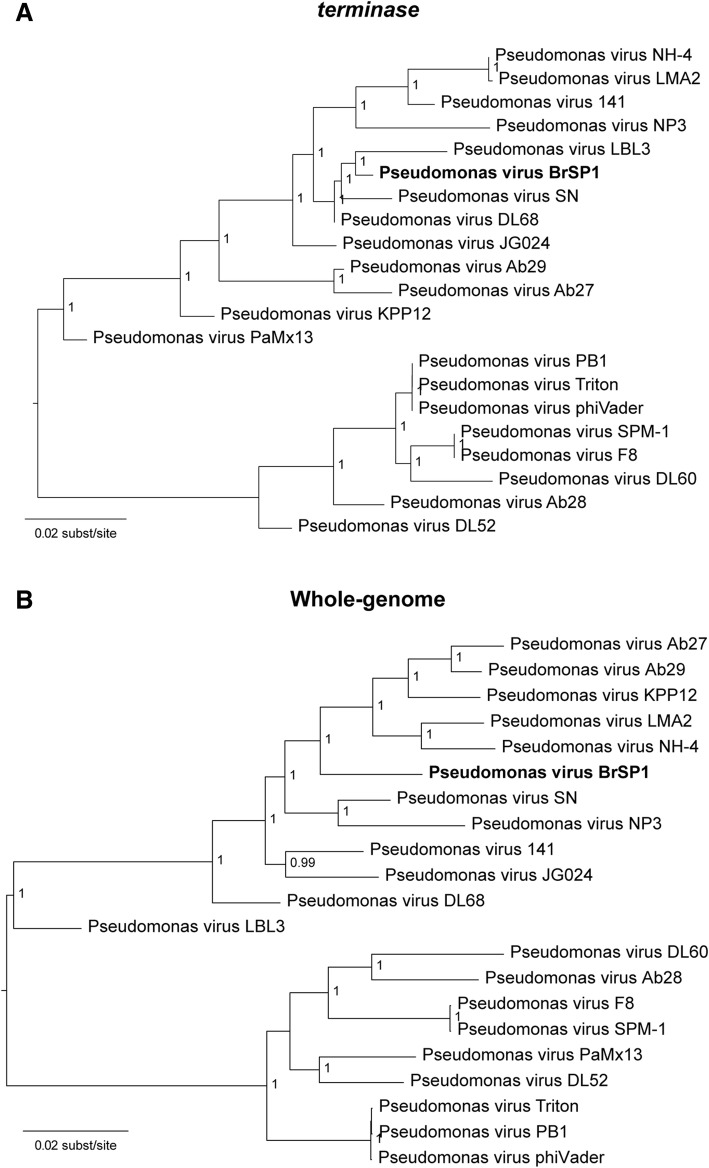
Phylogenetic relationships among pbunaviruses. Maximum likelihood (ML) trees were inferred by using FastTree, based on alignments of (**a**) *terminase* gene and (**b**) complete genome (without gaps) from pbunaviruses (21 isolates/species). The ICTV recognized species names are shown in italic and the Pseudomonas virus BrSP1 is shown in bold. The trees were midpoint rooted for clarity, and all horizontal branch lengths were drawn to a scale of nucleotide substitutions per site. Shimodaira-Hasegawa-like (SH-like) branch support values higher than 0.9 are shown

The terminase gene, which encodes an enzyme involved in the packaging of phage DNA into capsids, is a phylogenetic marker used in the investigation of several phage groups [51]. We inferred the phylogenetic relationship of phage BrSP1 to other pbunaviruses using both the terminase gene (Fig. 5a) and whole genome alignments (Fig. 5b). As shown in Fig. 5a, the phage BrSP1 terminase gene clustered together with a group of three ICTV recognized species (Pseudomonas virus LBL3, Pseudomonas virus SN, and Pseudomonas virus DL68), with a pairwise identity of 98, 99, and 99, respectively (data not shown). The phylogenetic tree inferred using whole-genome alignment (Fig. 5b) was partially incongruent with the terminase-based phylogeny (Fig. 5a), and phage BrSP1 was basal to the clade formed by Pseudomonas virus KPP12, Pseudomonas virus LMA12, Pseudomonas virus SN, Pseudomonas virus NH-4 and Pseudomonas virus Ab29 and Ab27.

## Discussion

The work here presented comprises the isolation and the characterization of the *Pseudomonas aeruginosa* BrSP1 bacteriophage. Our study involved the sequencing of the phage genome and the investigation of biological traits, such as host-range, “in vitro” biocontrol capacity, EOP estimation and the appearance of BIMs. These latter studies were done as a preliminary investigation to evaluate the potential of using BrSP1 in phage therapy against *P. aeruginosa* strains that infect domestic animals.

The sequencing of the BrSP1 genome revealed that its G + C content was 55.7%, which is very similar to those of other pbunaviruses [41] and lower than the GC content of *P. aeruginosa*, which is 66.6% [52]. Several other lytic phages that infect *P. aeruginosa* also display lower levels of GC content compared to the host [53–55]. Likewise, lytic phages that infect other members of the genus *Pseudomonas*, such as *P. fluorescens* [56–58] *P. putida* [59] and *P. stutzeri* [60], also have lower GC content levels compared to their host. Kwan and coworkers [61] hypothesized that differences in GC content between strains of *P. aeruginosa* and their phages could be due either to lateral gene transfer, to the recent invasion of these viruses to *P. aeruginosa* or some other characteristic feature of the viruses, such as variations on DNA polymerase and other replication related genes. Recent studies have shown that there is a relationship between the occurrence of mutator genes and genome GC variation [62, 63], suggesting that enzymes involved in replication and repair processes are the primary force diving GC content in bacteria.

The GC content of bacteria is considered a result of a complex interaction of endogenous and exogenous factors [64]. Analysis of genome databases has shown that the length of bacterial genomes is directly related to GC content [64]. Moreover, the presence of certain enzymes involved in replication and repair processes correlates with GC content [63, 65]. In their recent analysis, Almpanis and coworkers [64] concluded that phages, in general, have lower GC content than their hosts. The evolutionary advantage for lower GC content in these obligatory intracellular parasites is not well understood [64]. The higher energetic costs involved in the synthesis of GTP and CTP may be an influence [66]. Also, the fact that ATP pools are more abundant than that of other nucleotides may also affect this feature [66].

Our host range investigation, done mostly with *P. aeruginosa* strains isolated from domestic animals, showed that phage BrSP1 infected 51.4% of the strains tested (Table 1 and Additional file 2: Table S2). The susceptible strains included bacteria isolated from cows, dogs, and horses from different regions of Southern Brazil (Table 1 and Additional file 2: Table S2). The ability to infect a broad diversity of *P. aeruginosa* strains seems to be a trait of the pbunaviruses. Garbe and coworkers [42], for instance, found that phage JG024 infected 50% of 100 environmental strains collected from rivers and 84% of 19 clinical strains. Likewise, an investigation with phage KPP12 showed that this virus was able to infect 52.6% of 38 clinical strains [67].

Our EOP analysis revealed up to nearly one hundred fold EOP differences among the susceptible strains (Fig. 2). Changes on surface molecules that serve as bacteriophage receptors can significantly affect EOP and reduce phage adsorption [68–70]. It is, therefore, likely that the substantial EOP differences we found are a result of differences on the receptors of the strains. The majority of the susceptible strains were of medium or high efficiency according to the parameters proposed by Viazis and coworkers [71]. That is, their EOP values were above 0,2 [71]. Only three of the susceptible strains (CASM03, BOIJ03, and CASM11) displayed EOP values below 0,2 and were, thus, considered “low efficiency” (Fig. 2). The phage stock solution used in the EOP assay for these three strains had to be increased tenfold in order to produce lysis plaques. This need suggests that the use of phage BrSP1 as a control agent for the low-efficiency strains would require higher doses in order to be effective.

Our “in vitro” biological assays showed that bacterial growth remained checked for the first 12 h after phage infection, but that after 24 h there were little differences between infected and non-infected samples (Fig. 3). To find out the origin of this rapid development of resistance, we plated *P. aeruginosa* on a double layer agar plate which had phage BrSP1 on the overlay. The growth of colonies under these conditions showed that resistant variants arose independently of the presence of phage BrSP1 (Table 2). This result indicated that the growth observed at 24 h post-infection was attributed, at least partially, to the multiplication of resistant variants that occurred independently of the presence of the phage. Thus, these resistant bacteria were of the type Luria and Delbruck [72] termed “original variants.” Part of the resistant colonies that grew on the phage overlay produced a red pigment, similar to what Le and colleagues [69] observed in their study of phage-resistant *P. aeruginosa*. Their results also indicated that resistance arose spontaneously [69]. Other recent studies, both with *P. aeruginosa* and with other species, have investigated the spontaneous emergence of BIMs [68, 73, 74].

The development of resistance is a critical concern for phage therapy [75]. Thus, it is essential to consider the rapid recovery observed in our “in vitro” assays if phage BrSP1 is to be used to control *P. aeruginosa*. However, it is important to point out that the rapid development of resistance “in vitro” is not necessarily a barrier to the application of bacteriophages. Two recently published studies with *P. aeruginosa* support this assertion [76, 77]. Oeshslin and colleagues [76], for instance, showed that a combination of phages and antibiotics cleared heart valve infections in a rat model. Importantly, they investigated the growth of phage resistant bacteria in fibrin clots and heart tissues of infected rats. The rapid growth of resistant bacteria in the “in vitro” fibrin-clot model contrasted with the absence of BIMs in the tissues of the experimental animals [76]. Similarly, effective infection treatment in a cancer patient was observed using antibiotics and a phage cocktail which included phages that promoted rapid growth of resistant *P. aeruginosa* in broth cultures [77]. In both of the above cases, the “in vitro” assays displayed patterns similar to that observed in our investigation regarding the growth of phage-resistant variants (Fig. 3).

In fact, “in vitro” control patterns similar to that of BrSP1 has been observed in other studies done with Gram-negative bacteria [78, 79] (Jumpei Uchiyama, Ken Fukuda and Shigenobu Matsuzaki, personal communication, January, 2019) and the rapid regrowth of the bacterial populations seems to be a general phenomenon. The study with phages that infect *Vibrio anguillarum*, a pathogen of fish larvae, also showed that the fast recovery of the bacterial population “in vitro” is not necessarily an impediment to the successful application of a phage cocktail as a control measure [79].

Evidence from studies with other pbunaviruses indicates that resistance development is not a barrier for their application in phage therapy. Fukuda and coworkers [67], for instance, successfully treated keratitis in mice with a single application of Pseudomonas phage KPP12, a pbunavirus closely related to phage BrSP1 (Table 3). The treatment reduced bacterial load to almost undetectable levels and the cornea of treated mice had a remarkable improvement [67]. In another study, the use of a phage cocktail containing a pbunavirus and a podovirus practically eliminated *P. aeruginosa* from the lungs of infected mice [37].

The use of phage cocktails and the concomitant use of phages and antibiotics are strategies that can be used to reduce the growth of BIMs [76–78]. As changes in bacteriophage receptor is a common cause of resistance, the phages in the cocktail should ideally use different receptor molecules [68]. Crucially, mutations that render the bacteria resistant to bacteriophages often reduce the fitness of the new variants [68, 69]. This fitness cost may be exploited to promote the successful application of phage therapy [75].

## Conclusion

In this work, we have isolated and characterized a *P. aeruginosa* bacteriophage, which we named Pseudomonas phage BrSP1. The virus was able to infect 51.4% of our collection of *P. aeruginosa* strains. The susceptible strains were clinical isolates from domestic animals and the reference strain ATCC 27853. “In vitro” bioassays using phage BrSP1 resulted in a rapid reduction of bacterial load. These results indicated that phage BrSP1 is a broad host range virus that could be included in a phage cocktail to treat *P. aeruginosa* infections in domestic animals. The efficiency of plating (EOP) values for phage BrSP1 varied considerably among the tested strains. We suggest the use of publicly available strains as the reference in EOP analysis, as it allows comparison of results obtained in different laboratories. Based on comparisons of complete genome sequences, we propose that Pseudomonas phage BrSP1 is a novel species of the genus *Pbunavirus*. In our phylogenetic analysis, we found topological incongruences between the commonly used *terminase* gene and the tree constructed using the complete genome. The complete genome of BrSP1 phage provides additional data that may help the broader understanding of pbunaviruses genome evolution.

## Methods

### *P. aeruginosa* origin and maintenance

Except for *P. aeruginosa* Lfar01 and *P. aeruginosa* ATCC 27853, the strains used in this study were isolated from samples sent from various sources for microbiological analysis at Dr. Agueda Vargas laboratory (Department of Preventive Veterinary Medicine, Federal University of Santa Maria, Santa Maria, Brazil; Additional file 2: Table S2). The bacteria isolated from animals are registered in the SISGEN platform under the registration code A28EE0E. SISGEN (Sistema Nacional de Gestão do Patrimônio Genético e do Conhecimento Tradicional Associado) is a national registration system of Brazilian biological material. Samples were cultivated on blood agar containing 5% sheep blood and on MacConkey agar (Difco). Cultures were incubated aerobically for 24–48 h at 36 °C. After incubation, isolates were identified according to conventional microbiological methods [80]. The origin of strain Lfar01 is unknown. *P. aeruginosa* was propagated in Tryptic Soy Broth (TSB) and Agar (TSA) (Fluka Analytical). TSA supplemented with 15% glycerol was used for long-term storage at − 80 °C.

### 16S rRNA gene sequencing

Sequencing analysis of the 16S rRNA genes were done for the strain Lfar01 and for those isolates that were not susceptible to BrSP1 infection. DNA extraction was done with the kit Brazol (LGC Biotecnologia), according to the manufacturer’s instructions. Primers 27f (5′-CAGGCC TAA CAC ATG CAA GTC-3′) and 1387r (5′-GGG CGG WGT GTA CAA GGC-3′) [81] were used for the amplification and the sequencing reaction of the purified amplicon. The reactions were carried out in a T100™ Thermal Cycler (Bio-Rad®) following the conditions described by Marchesi, et al. [81]. The PCR product was purified using E.Z.N.A.® Cycle-Pure Kit 200 (Omega Bio-Tek®) and sequenced with the BigDye Terminator Kit® (v. 3.1 RR-100) according to the manufacture’s instruction (PE Applied Biosystems). Electrophoresis was carried out on ABI 3130 Genetic Analyzer (Applied biosystems) at Genomic Engenharia Molecular Ltda (São Paulo, Brazil). The sequences obtained were assembled in the CodonCode Aligner, version 3 (CodonCode Corporation) and compared with those of the Genbank using the Blast program [49].

### Phage isolation

Phage BrSP1 was isolated from samples of untreated sewage collected at SABESP Pinheiros (Water treatment utility located in Sao Paulo, Brazil). Six samples of 50 mL were transferred to conical centrifuge tubes and subjected to centrifugation (centrifuge Hitachi®, model CR22G) at 4000 x g for 5 min. A total of 45 mL of supernatant was transferred to borosilicate flasks containing 45 mL of doubled concentrated TSB media. Approximately 5 mL of *P. aeruginosa* strain Lfar01 (Additional file 2: Table S2) at exponential growth phase was added to the flask, and incubated at 36 °C in a benchtop incubator shaker (model Classic C24, New Brunswick Scientific®) at 120 rpm for 24 h. After this period, a 10 mL sample was removed from the flask, transferred to a 15 mL conical tube and centrifuged for 10 min at 4000 x g (centrifuge Hitachi®, model CR22G). Approximately 5 mL of the supernatant were removed and passed through a sterile polyvinylidene fluoride syringe filter (JET BIOFIL®) with pore size of 0.22 μm.

Phage presence was evaluated using the small drop assay. The double agar method [82] was used to prepare the bacterial lawn. TSA media was plated in the bottom layer and the top layer was prepared adding 0.25% of agarose (Agargen®) to 5 mL of TSB. The agarose was dissolved by heating the broth in a microwave oven and then kept at 50 °C in a water bath. Approximately 1 mL of *P. aeruginosa* at exponential growth phase was added to the TSB/agarose mixture immediately before pouring it over the bottom layer. Aliquots of 20 μL of each of the six samples, processed as described above, were spotted on the top layer. The agar plates were incubated for 24 h at 36 °C. After this period, the plates were checked for the presence of clear lysis zones, indicating the presence of bacteriophages.

### Phage purification, titration of phage stocks, storage stability and electron microscopy

The plaque purification method described by Miller [83] was used to obtain a homogeneous phage stock. The viral sample that produced plaques was serially diluted using SM buffer (100 mM NaCl; 8 mM MgSO4•7H2O; 50 mM Tris-Cl pH 7.5; 0.002% gelatin) up to 10 million fold. A total of 20 μL of the serially diluted phage was added to 200 μL of *P. aeruginosa* cells at stationary phase of growth. The samples were homogenized, kept at room temperature for 20 min., mixed to 6 mL of molten TSB supplement with salts and 0.25% agarose, poured over a TSA plate, and incubated for a period of 48 h. at 36 °C.

After the incubation period, a slab of the double layer with a single plaque was removed from the plate with a blade and transferred to a microtube containing 1 mL of SM buffer. The microtube was subjected to agitation in a benchtop shaker (Quimis®) for 1 h at 500 rpm. Phage released in the buffer was then purified with 200 μL chloroform. After 10 min of agitation, the sample was centrifuged for 10 min at 5000 x g (Centrifuge 5418, Eppendorf®). The supernatant was transferred to a new microtube. The whole process, starting from the serial dilution, was repeated twice. After the third round of purification, 10 μL of the chloroform-purified sample was used to infect 10 mL of *P. aeruginosa* cells at early exponential growth phase. The infected cells were incubated at 36 °C in a benchtop incubator shaker (model Classic C24, New Brunswick Scientific®) at 120 rpm for 24 h. The culture was then centrifuged and the supernatant passed through a sterile Polyvinylidene fluoride syringe filter (JET BIOFIL®) with pore size of 0.22 μm. This was the phage stock that was used in the subsequent analysis. The titer of this stock was evaluated. For that, three series of serial dilutions were done with a sample of the stock. The serially diluted phages were used to infect cells as described above. Plaques formed at specific dilutions were counted and used to estimate the plaque forming units per mL.

We investigated the storage stability of phage BrSP1 under refrigeration (approximately 5 °C). We estimated the titer of a BrSP1 stock in triplicates before and after storage for 104 days in the refrigerator (Additional file 6).

We also examined Phage BrSP1 by electron microscopy. Two hundred microliters of a high titer chloroform purified phage stock were placed on a Parafilm® surface. Copper grids (200 mesh) covered with carbon-coated Formvar film were floated on them for 10 min, washed with droplets of distilled water and floated on droplets of 1% aqueous uranyl acetate for 10 min. After that, grids were removed and excess of liquid eliminated with a filter paper. These negatively stained preparations were examined in a JEOL JEM 1011 transmission electron microscope (installed at the Laboratório de Microscopia Eletrônica, Departamento de Fitopatologia e Entomologia, Escola Superior de Agricultura Luiz de Queiroz, Universidade de São Paulo, campus Piracicaba) at 60 KV and images registered digitally.

### Host range investigation

In total, 36 *P. aeruginosa* strains isolated from domestic animals and *P. aeruginosa* strain ATCC 27853 were investigated regarding their susceptibility to bacteriophage BrSP1. Spot test on a double agar overlay [82] was used in this analysis. Plates contained each of the 37 bacterial isolates on the top layer were prepared adding 1 mL of a culture at early exponential phase to 5 mL of molten TSB media and 0.25% agarose (Agargen). A first round of analysis was done with the undiluted phage stock. Aliquots of 20 μL of phage stock containing approximately 4.5 × 10^5^ PFU were spotted with a precision pipette on the surface of the agar overlay. The plates were incubated for a period of up to 48 h at 36 °C. After this period, the plates were checked for the appearance of lytic inhibition zones, which were classified as clear, turbid or not present.

Bacterial isolates that displayed clear or turbid zones of inhibition on this test were used in a second round of analysis following the same procedure described above, except that serial diluted phage stock was used (up to 10^6^ dilutions). Again, the plates were incubated for a period of up to 48 h at 36 °C. This time, the plates were checked for the appearance of individual inhibition plaques.

### Efficiency of plating (EOP) analysis

For the efficiency of plating (EOP) evaluation, the double agar plate assays were done according to the procedure described by Mirzaei and Nilsson [84]. The phage stock used in these experiments was titrated in triplicates using *P. aeruginosa* strain ATCC 27853. A volume of 10 μL of diluted phage stock, containing approximately 24 PFU, was added to 200 μL of susceptible *P. aeruginosa* culture at the stationary phase of growth. The samples were homogenized, kept at room temperature for 20 min., mixed to 6 mL of molten TSB and 0.25% agarose, poured over a TSA plate, and incubated for a period of up to 48 h at 36 °C. The number of lysis plaques formed was recorded after this period. A few samples did not display lysis plaques with the standard phage inoculum used in the assay. For those samples, the experiment was redone using a phage stock that was 10 times larger. EOP was estimated as the ratio of the number of lysis plaques produced in the *P. aeruginosa* strain ATCC 27853 to the number of plaques produced in each of the other strains analyzed (Additional file 7). These assays were done in triplicates.

### Biological assays

In order to evaluate the capacity of phage BrSP1 to control *P. aeruginosa*, we carried out “in vitro” experiments with strains Lfar01, ATCC 27853 and BOIJ02. TSB medium supplemented with CaCl2 (40 mg/L) and MgSO4 (40 mg/L) was used in these analyses. The phage concentration we used were either 10^6^ or 10^7^ plaque forming units per mL of bacterial culture media (PFU/mL). Test tubes with 10 mL of *P. aeruginosa* cells at early exponential phase were inoculated with phage BrSP1 and cultured at 36 °C in a benchtop incubator shaker (model Classic C24, New Brunswick Scientific®) at 120 rpm for 24 h. We removed samples from the culture at the time of viral inoculation (t = 0) and at 2, 4, 6, 8, 10, 12, and 24 h post-infection (h p.i.) (t = 2, t = 4, t = 6, t = 8, t = 10, t = 12, and t = 24, respectively). Control tubes were inoculated with SM buffer. We evaluated cell concentrations at each of these times measuring the absorbance at 600 nm (OD_600_) (NanoDrop model 2000c, Thermo Scientific®) (Additional file 8). Each set of assays was done in triplicate.

### Frequency of occurrence of phage resistant mutants

We investigated the frequency of phage-resistant mutants in *P. aeruginosa* strains Lfar01, ATCC 27853 and BOIJ02 following the method employed by Filippov and colleagues [68]. We inoculated the strains on TSA plates using the steak plate technique to isolate individual colonies. We inoculated bacterial cells from 5 colonies of each strain to a test tube containing 5 mL of TSA. After overnight growth at 36 °C, samples of the bacterial cultures were serially diluted. These samples were inoculated on TSA plates with or without a double layer containing approximately 1 × 10^9^ PFU of phage BrSP1. One hundred microliters of undiluted bacterial culture and of dilutions up to 10^− 2^ were spread plated on the surfaces of TSA plates containing phages on the top layer. The same volumes of dilutions 10^− 7^ to 10^− 9^ were spread plated on TSA plates without phages. Plates were incubated for a period of up to 48 h at 36 °C. Individual colonies were counted and the concentration of phage resistant bacteria and total bacteria was estimated in terms of colony forming units per mL (CFU/mL). The frequency of spontaneous resistant mutations was estimated as the ratio between the numbers of resistant to total bacterial counts per mL of the overnight culture (Additional file 9).

### Phage DNA extraction

Phage DNA extraction was done following the procedure described by Su et al. [85]. A total of 10 mL of *P. aeruginosa* cells (strain Lfar01) at exponential growth phase were inoculated with 50 μL of diluted phage stock. The inoculated cells were cultured at 36 °C in a benchtop incubator shaker (model Classic C24, New Brunswick Scientific®) at 120 rpm for 24 h. The culture was then transferred to a 15 mL conical tube and centrifuged (centrifuge Hitachi®, model CR22G) at 4000 x g for a period of 5 min. The supernatant was transferred to a new tube and treated with 10 units of amplification grade DNase I (Sigma-Aldrich) for a period of 30 min. at 36 °C. Following this treatment, 200 μL of a 2 M ZnCl_2_ (filter sterilized) solution, which promotes phage particles precipitation, was added. The mixture was allowed to stand for 5 min at 36 °C and was centrifuged (centrifuge Hitachi®, model CR22G) at 4000 x g for a period of 5 min. The phage pellet was dissolved in TENS buffer (50 mM Tris-HCl, pH 8, 100 mM EDTA, 100 mM NaCl and 0.3% SDS) and proteinase K was added to a final concentration of 100 μg/mL. The solution was vigorously mixed and incubated at 65 °C overnight. The DNA was then extracted using a phenol/chloroform/isoamyl alcohol mixture and ethanol precipitated. The DNA was resuspended in 100 μL of ultrapure water.

### Sequencing, assembly and analysis of the phage BrSP1 genome

The sequencing library was prepared using TruSeq Nano DNA Kit and sequenced with HiSeq 2500 System (2 × 100 paired-end) at Macrogen Inc. (Seoul, Republic of Korea). The resulting reads were filtered and trimmed to remove short and low-quality regions/reads and then assembled using CLC Genomics Workbench (CLC Bio, Aarhus, Denmark) with default parameters. The read mapping and genome annotation was performed using Geneious 7.1.8 [86]. ORFs were annotated using BLASTx search against the NCBI non-redundant protein database. Maximum likelihood (ML) tree reconstruction was performed with FastTree using the default options [87] on alignments of *terminase* genes and complete genomes (without gaps) from pbunaviruses (21 isolates/species). The branch support values were estimated with the Shimodaira-Hasegawa test [88]. The pairwise genome comparison was performed using PASC [50]. The genome of selected species and strains were compared using MAUVE [89] and plotted using genoPlotR package [90] available for R.

## Additional files

Additional file 1: **Table S1.** Storage stability of phage BrSP1 under refrigerated temperature. (DOCX 11 kb)

Additional file 2: **Table S2.** List of *P. aeruginosa* strains used in this study. (DOCX 15 kb)

Additional file 3: **Table S3.** Genbank accession number of the 16S rRNA genes of strains that were not susceptible to phage BrSP1 (DOCX 12 kb)

Additional file 4: **Table S4.** ORFs identified in the genome of Pseudomonas phage BrSP1 (DOCX 41 kb)

Additional file 5: **Table S5.** Codon usage in phage BrSP1 and *in P. aeruginosa* (PAO) (DOCX 18 kb)

Additional file 6: Titers of phage before and after refrigeration for 104 days. (XLSX 13 kb)

Additional file 7: Estimation of EOP. (XLSX 20 kb)

Additional file 8: Absorbance data collected in the experiments. (XLSX 19 kb)

Additional file 9: Frequency of spontaneous BIMs. (XLSX 16 kb)

## Abbreviations

ATCC: American Type Culture Collection
BIMs: Bacteriophage Insensitive Mutants
CFU: Colony forming units
EOP: Efficiency of plating
MOI: Multiplicity of infection
PCR: Polimerase Chain Reaction
PFU: Plaque forming units

## References

[1] Davies JC (2002). *Pseudomonas aeruginosa* in cystic fibrosis: pathogenesis and persistence. Paediatr Respir Rev.

[2] Altoparlak U, Erol S, Akcay MN, Celebi F, Kadanali A (2004). The time-related changes of antimicrobial resistance patterns and predominant bacterial profiles of burn wounds and body flora of burned patients. Burns..

[3] Church D, Elsayed S, Reid O, Winston B, Lindsay R (2006). Burn wound infections. Clin Microbiol Rev.

[4] Gaynes R, Edwards JR (2005). National Nosocomial Infections Surveillance System. Overview of nosocomial infections caused by gram-negative bacilli. Clin Infect Dis.

[5] Weiner LM, Webb AK, Limbago B, Dudeck MA, Patel J, Kallen AJ (2016). Antimicrobial-Resistant Pathogens Associated with Healthcare-Associated Infections: Summary of Data Reported to the National Healthcare Safety Network at the Centers for Disease Control and Prevention, 2011-2014. Infect Control Hosp Epidemiol.

[6] Ringen LM, Drake CH (1952). A study of the incidence of *Pseudomonas aeruginosa* from various natural sources. J Bacteriol.

[7] Green SK, Schroth MN, Cho JJ, Kominos SK, Vitanza-Jack VB (1974). Agricultural plants and soil as a reservoir for *Pseudomonas aeruginosa*. Appl Microbiol.

[8] Deredjian A, Colinon C, Hien E (2014). Low occurrence of *Pseudomonas aeruginosa* in agricultural soils with and without organic amendment. Front Cell Infect Microbiol.

[9] Estepa V, Rojo-Bezares B, Torres C, Sáenz Y (2014). Faecal carriage of Pseudomonas aeruginosa in healthy humans: antimicrobial susceptibility and global genetic lineages. FEMS Microbiol Ecol.

[10] DWF W, Mara DD, Jawad L (1980). Oragui, J Pseudomonas aeruginosa and Escherichia coli in sewage and fresh water. Water Res.

[11] Mena KD, Gerba CP (2009). Risk assessment of Pseudomonas aeruginosa in water. Rev Environ Contam Toxicol.

[12] Correa C.M.C., Tibana A., Filho P.P.Gontijo (1991). Vegetables as a source of infection with Pseudomonas aeruginosa in a University and Oncology Hospital of Rio de Janeiro. Journal of Hospital Infection.

[13] Apidianakis Y, Rahme LG (2009). Drosophila melanogaster as a model host for studying *Pseudomonas aeruginosa* infection. Nat Protoc.

[14] Tan MW, Mahajan-Miklos S, Ausubel FM (1999). Killing of Caenorhabditis elegans by *Pseudomonas aeruginosa* used to model mammalian bacterial pathogenesis. Proc Natl Acad Sci.

[15] Thomas J, Thanigaivel S, Vijayakumar S, Acharya K, Shinge D, Seelan TSJ (2014). Pathogenecity of *Pseudomonas aeruginosa* in Oreochromis mossambicus and treatment using lime oil nanoemulsion. Colloids Surf B: Biointerfaces.

[16] Walker SE, Sander JE, Cline JL, Helton JS (2002). Characterization of *Pseudomonas aeruginosa* isolates associated with mortality in broiler chicks. Avian Dis.

[17] Allen JL, Begg AP, Browning GF (2011). Outbreak of equine endometritis caused by a genotypically identical strain of *Pseudomonas aeruginosa*. J Vet Diagn Investig.

[18] Kidd TJ, Gibson JS, Moss S, Greer RM, Cobbold RN, Wright JD, Ramsay KA, Grimwood K, Bell SC (2011). Clonal complex *Pseudomonas aeruginosa* in horses. Vet Microbiol.

[19] Barkema HW, Schukken YH, Lam TJGM, Beiboer ML, Wilmink H, Benedictus G (1998). Incidence of clinical mastitis in dairy herds grouped in three categories by bulk Milk somatic cell counts. J Dairy Sci.

[20] Bhatt VD, Ahir VB, Koringa PG, Jakhesara SJ, Rank DN, Nauriyal DS, Kunjadia AP, Joshi CG (2012). Milk microbiome signatures of subclinical mastitis-affected cattle analysed by shotgun sequencing. J Appl Microbiol.

[21] Park H, Hong M, Hwang S, Park Y, Kwon K, Yoon J (2014). Characterisation of *Pseudomonas aeruginosa* related to bovine mastitis. Acta Vet Hung.

[22] Hirakawa Y, Sasaki H, Kawamoto E, Ishikawa H, Matsumoto T, Aoyama N (2010). Prevalence and analysis of *Pseudomonas aeruginosa* in chinchillas. BMC Vet Res.

[23] Hammer AS, Pedersen K, Andersen TH, Jørgensen JC, Dietz HH (2003). Comparison of *Pseudomonas aeruginosa* isolates from mink by serotyping and pulsed-field gel electrophoresis. Vet Microbiol.

[24] Salomonsen CM, Themudo GE, Jelsbak L, Molin S, Høiby N, Hammer AS (2013). Typing of Pseudomonas aeruginosa from hemorrhagic pneumonia in mink (Neovison vison). Vet Microbiol.

[25] Qi J, Li L, Du Y, Wang S, Wang J, Luo Y (2014). The identification, typing, and antimicrobial susceptibility of *Pseudomonas aeruginosa* isolated from mink with hemorrhagic pneumonia. Vet Microbiol.

[26] Gatoria IS, Saini NS, Rai TS, Dwivedi PN (2006). Comparison of three techniques for the diagnosis of urinary tract infections in dogs with urolithiasis. J Small Anim Pract.

[27] Hariharan H, Coles M, Poole D, Lund L, Page R (2006). Update on antimicrobial susceptibilities of bacterial isolates from canine and feline otitis externa. Can Vet J.

[28] Hillier A, Alcorn J, Cole L (2006). Pyoderma caused by *Pseudomonas aeruginosa* infection in dogs: 20 cases. Vet Dermatol.

[29] Mekić S, Matanović K, Šeol B (2011). Antimicrobial susceptibility of *Pseudomonas aeruginosa* isolates from dogs with otitis externa. Vet Rec.

[30] Thompson MF, Litster AL, Platell JL, Trott DJ (2011). Canine bacterial urinary tract infections: new developments in old pathogens. Vet J.

[31] Ciocan OA, Carp-Carare M, Rimbu C, Cozma AP, Carp-Carare C, Guguianu E (2015). The incidence of dog recurrent otitis caused by strains of multidrug-resistant (MDR) *Pseudomonas aeruginosa*. Lucr Stiint Stiinte Agric a Banat Timisoara. Med Vet.

[32] Silva LCA, Pessoa DAN, Maia LA, Matos RATM, Macêdo MMS (2016). Systemic infection by *Pseudomonas aeruginosa* in a dog. Acta Sci Vet.

[33] May TB, Roychoudhury S, Zielinski NA, Berry A, Rothmel RK, Shinabarger D (1991). Alginate synthesis by *Pseudomonas aeruginosa*: a key pathogenic factor in chronic pulmonary infections of cystic fibrosis patients. Clin Microbiol Rev.

[34] Costerton J, Lewandowski Z, Caldwell D, Korber D, Lappin-Scott H (1995). Microbial biofilms. Annu Rev Microbiol.

[35] Harper DR, Enright MC (2011). Bacteriophages for the treatment of *Pseudomonas aeruginosa* infections. J Appl Microbiol.

[36] Pires DP, Vilas Boas D, Sillankorva S, Azeredo J (2015). Phage therapy: a step forward in the treatment of *Pseudomonas aeruginosa* infections. J Virol.

[37] Alemayehu D, Casey PG, Mcauliffe O (2012). Bacteriophages φMR299-2 and φNH-4 can eliminate *Pseudomonas aeruginosa* in the murine lung and on cystic fibrosis lung airway cells. MBio..

[38] Rose T, Verbeken G, Vos DD, Merabishvili M, Vaneechoutte M, Jennes S (2014). Experimental phage therapy of burn wound infection : difficult first steps. Int J Burn Trauma.

[39] Santos TMA, Ledbetter EC, Caixeta LS, Bicalho MLS, Bicalho RC (2011). Isolation and characterization of two bacteriophages with strong in vitro antimicrobial activity against *Pseudomonas aeruginosa* isolated from dogs with ocular infections. Am J Vet Res.

[40] Hawkins C, Harper D, Burch D, Änggård E, Soothill J (2010). Topical treatment of *Pseudomonas aeruginosa* otitis of dogs with a bacteriophage mixture: a before/after clinical trial. Vet Microbiol.

[41] Ceyssens PJ, Miroshnikov K, Mattheus W, Krylov V, Robben J, Noben JP (2009). Comparative analysis of the widespread and conserved PB1-like viruses infecting *Pseudomonas aeruginosa*. Environ Microbiol.

[42] Garbe J, Wesche A, Bunk B, Kazmierczak M, Selezska K, Rohde C (2010). Characterization of JG024, a *Pseudomonas aeruginosa* PB1-like broad host range phage under simulated infection conditions. BMC Microbiol.

[43] Abedon ST (2011). Lysis from without. Bacteriophage..

[44] Kutter E (2009). Phage host range and efficiency of plating. Methods Mol Biol.

[45] Sharp PM, Li W (1986). Codon usage in regulatory genes in Escherichia coli does not reflect selection for 'rare' codons. Nucleic Acids Res.

[46] Grocock RJ, Sharp PM (2002). Synonymous codon usage in *Pseudomonas aeruginosa* PA01. Gene..

[47] Mavrich TN, Hatfull GF (2017). Bacteriophage evolution differs by host, lifestyle and genome. Nat Microbiol.

[48] Adriaenssens Evelien, Brister J. Rodney (2017). How to Name and Classify Your Phage: An Informal Guide. Viruses.

[49] Altschul SF, Gish W, Miller W, Myers EW, Lipman DJ (1990). Basic local alignment search tool. J Mol Biol.

[50] Bao Y, Chetvernin V, Tatusova T (2014). Improvements to pairwise sequence comparison (PASC): a genome-based web tool for virus classification. Arch Virol.

[51] Serwer P, Hayes SJ, Zaman S, Lieman K, Rolando M, Hardies SC (2004). Improved isolation of undersampled bacteriophages: finding of distant terminase genes. Virology..

[52] Stover CK, Pham XQ, Erwin AL, Mizoguchi SD, Warrener P, Hickey MJ (2000). Complete genome sequence of *Pseudomonas aeruginosa* PAO1, an opportunistic pathogen. Nature..

[53] Sepúlveda-Robles O, Kameyama L, Guarneros G (2012). High diversity and novel species of *Pseudomonas aeruginosa* bacteriophages. Appl Environ Microbiol.

[54] Cruz-Plancarte I, Cazares A, Guarneros G (2016). Genomic and transcriptional mapping of PaMx41, archetype of a new lineage of bacteriophages infecting *Pseudomonas aeruginosa*. Appl Environ Microbiol.

[55] Flores V, Sepúlveda-Robles O, Cazares A, Kameyama L, Guarneros G (2017). Comparative genomic analysis of *Pseudomonas aeruginosa* phage PaMx25 reveals a novel Siphovirus group related to phages infecting hosts of different taxonomic classes. Arch Virol.

[56] Eller MR, Salgado RL, Vidigal PMP, Alves MP, Dias RS, de Oliveira LL (2013). Complete Genome Sequence of the *Pseudomonas fluorescens* Bacteriophage UFV-P2. Genome Announc.

[57] Wojtus JK, Fitch JL, Christian E, Dalefield T, Lawes JK, Kumar K, Peebles CL, Altermann E, Hendrickson HL (2017). Complete Genome Sequences of Three Novel *Pseudomonas fluorescens* SBW25 Bacteriophages, Noxifer, Phabio, and Skulduggery. Genome Announc.

[58] Lu G, Luhr J, Stoecklein A, Warner P, Tapprich W (2017). Complete Genome Sequences of *Pseudomonas fluorescens* Bacteriophages Isolated from Freshwater Samples in Omaha, Nebraska. Genome Announc.

[59] Kovalyova IV, Kropinski AM (2003). The complete genomic sequence of lytic bacteriophage gh-1 infecting *Pseudomonas putida* - evidence for close relationship to the T7 group. Virology..

[60] Sillankorva S, Kropinski AM, Azeredo J (2012). Genome sequence of the broad-host-range *Pseudomonas* phage Φ-S1. J Virol.

[61] Kwan T, Liu J, Dubow M, Gros P, Pelletier J (2006). Comparative genomic analysis of 18 *Pseudomonas aeruginosa* bacteriophages. J Bacteriol.

[62] Lind PA, Andersson DI (2008). Whole-genome mutational biases in bacteria. Proc Natl Acad Sci U S A.

[63] Wu Hao, Zhang Zhang, Hu Songnian, Yu Jun (2012). On the molecular mechanism of GC content variation among eubacterial genomes. Biology Direct.

[64] Almpanis A, Swain M, Gatherer D, McEwan N. Correlation between bacterial G+C content, genome size and the G+C content of associated plasmids and bacteriophages. [published online ahead of print, 2018 Apr 10]. Microb Genom 2018;4(4). 10.1099/mgen.0.000168 PMC5989581 29633935

[65] Timinskas K, Balvočiūtė M, Timinskas A, Venclovas Č (2013). Comprehensive analysis of DNA polymerase III α subunits and their homologs in bacterial genomes. Nucleic Acids Res.

[66] Rocha EP, Danchin A (2002). Base composition bias might result from competition for metabolic resources. Trends Genet.

[67] Fukuda K, Ishida W, Uchiyama J, Rashel M, Kato S, Morita T, Muraoka A, Sumi T, Matsuzaki S, Daibata M, Fukushima A (2012). Pseudomonas aeruginosa keratitis in mice: effects of topical bacteriophage KPP12 administration. PLoS One.

[68] Filippov AA, Sergueev KV, He Y (2011). Bacteriophage-resistant mutants in Yersinia pestis: identification of phage receptors and attenuation for mice. PLoS One.

[69] Le S, Yao X, Lu S, Tan Y, Rao X, Li M, Jin X, Wang J, Zhao Y, Wu NC, Lux R, He X, Shi W, Hu F (2014). Chromosomal DNA deletion confers phage resistance to Pseudomonas aeruginosa. Sci Rep.

[70] Denes T, den Bakker HC, Tokman JI, Guldimann C, Wiedmann M (2015). Selection and characterization of phage-resistant mutant strains of listeria monocytogenes reveal host genes linked to phage adsorption. Appl Environ Microbiol.

[71] Viazis S, Akhtar M, Feirtag J, Brabban AD, Diez-Gonzalez F (2011). Isolation and characterization of lytic bacteriophages against enterohaemorrhagic Escherichia coli. J Appl Microbiol.

[72] Luria SE, Delbrück M (1943). Mutations of Bacteria from Virus Sensitivity to Virus Resistance. Genetics.

[73] Wittmann J, Dreiseikelmann B, Rohde C, Rohde M, Sikorski J (2014). Isolation and characterization of numerous novel phages targeting diverse strains of the ubiquitous and opportunistic pathogen Achromobacter xylosoxidans. PLoS One.

[74] Wright RCT, Friman VP, Smith MCM, Brockhurst MA (2018). Cross-resistance is modular in bacteria-phage interactions. PLoS Biol.

[75] Oechslin Frank (2018). Resistance Development to Bacteriophages Occurring during Bacteriophage Therapy. Viruses.

[76] Oechslin F, Piccardi P, Mancini S, Gabard J, Moreillon P, Entenza JM, Resch G, Que YA (2017). Synergistic Interaction Between Phage Therapy and Antibiotics Clears Pseudomonas Aeruginosa Infection in Endocarditis and Reduces Virulence. J Infect Dis.

[77] Ferry T, Boucher F, Fevre C, Perpoint T, Chateau J, Petitjean C, Josse J, Chidiac C, L'hostis G, Leboucher G, Laurent F, Lyon bone and joint infection study group (2018). Innovations for the treatment of a complex bone and joint infection due to XDR Pseudomonas aeruginosa including local application of a selected cocktail of bacteriophages. J Antimicrob Chemother.

[78] O'Flynn G, Coffey A, Fitzgerald GF, Ross RP (2006). The newly isolated lytic bacteriophages st104a and st104b are highly virulent against salmonella enterica. J Appl Microbiol.

[79] Silva YJ, Costa L, Pereira C, Mateus C, Cunha A, Calado R, Gomes NC, Pardo MA, Hernandez I, Almeida A (2014). Phage therapy as an approach to prevent Vibrio anguillarum infections in fish larvae production. PLoS One.

[80] Quinn PJ, Markey B, Carter ME, Carter GR (1994). Clinical veterinary microbiology.

[81] Marchesi JR, Sato T, Weightman AJ, Martin A, Fry JC, Hiom SJ, et al. Design and Evaluation of Useful Bacterium-Specific PCR Primers That Amplify Genes Coding for Bacterial 16S rRNA. Appl Environ Microbiol 1998;64(2):795–9. 10.1128/aem.64.2.795-799.1998 PMC106123 9464425

[82] Adams MH (1959). Assay of phages by the agar layer method. Bacteriophages.

[83] Miller RV, Burlage RS, Atlas R, Stahl D, Geesey G, Sayer GS (1998). Methods for enumeration and characterization of bacteriophages from environment samples. Techniques in microbial ecology.

[84] Mirzaei MK, Nilsson AS (2015). Isolation of phages for phage therapy: a comparison of spot tests and efficiency of plating analyses for determination of host range and efficacy. PLoS One.

[85] Su MT, Venkatesh TV, Bodmer R (1998). Large- and small-scale preparation of bacteriophage lambda lysate and DNA. Biotechniques.

[86] Kearse M, Moir R, Wilson A, Stones-Havas S, Cheung M, Sturrock S (2012). Geneious basic: an integrated and extendable desktop software platform for the organization and analysis of sequence data. Bioinformatics.

[87] Price MN, Dehal PS, Arkin AP (2010). FastTree 2 - approximately maximum-likelihood trees for large alignments. PLoS One.

[88] Shimodaira H, Hasegawa M (1999). Multiple comparisons of log-likelihoods with applications to phylogenetic inference. Mol Biol Evol.

[89] Darling ACE, Mau B, Blattner FR, Perna NT (2004). Mauve: multiple alignment of conserved genomic sequence with rearrangements. Genome Res.

[90] Guy L, Kultima JR, Andersson SGE, Quackenbush J (2010). GenoPlotR: comparative gene and genome visualization in R. Bioinformatics..

