# Supplementary material for: Characterization of a bacteriophage with broad host range against strains of Pseudomonas aeruginosa isolated from domestic animals
Source: BMC Microbiol. 2019 Jun 17;19:134. doi: 10.1186/s12866-019-1481-z (PMC6580649; doi:10.1186/s12866-019-1481-z)
Supplement: Supplementary file 1 — Table S1. Storage stability of phage BrSP1 under refrigerated temperature. (DOCX 11 kb) [file 12866_2019_1481_MOESM1_ESM.docx]

**Additional file 1: Table S1.**  Storage stability of phage BrSP1 under refrigerated temperature.

| **Day of phage stock titration** | **Mean titer ^1^** | **Standard Deviation** |
| --- | --- | --- |
| 1 | 8,50E+09 | 4,36E+09 |
| 104 | 6,83E+08 | 1,44E+08 |

^1^ Average of three independent experiments.
