# Supplementary material for: Characterization of a bacteriophage with broad host range against strains of Pseudomonas aeruginosa isolated from domestic animals
Source: BMC Microbiol. 2019 Jun 17;19:134. doi: 10.1186/s12866-019-1481-z (PMC6580649; doi:10.1186/s12866-019-1481-z)
Supplement: Supplementary file 2 — Table S2. List of P. aeruginosa strains used in this study. (DOCX 15 kb) [file 12866_2019_1481_MOESM2_ESM.docx]

**Additional file 2: Table S2.** List of *P. aeruginosa* strains used in this study.

| **Isolate name** | **Sample number** | **Specie of origin** | **Place of Origin ^1^** |
| --- | --- | --- | --- |
| Lfar01 | **0** | Unknown | Unknown |
| SWSM01 | **1.1** | Swine | Santa Maria, RS |
| SWSM02 | **1.2** | Swine | Santa Maria, RS |
| SWSM03 | **2.1** | Swine | Santa Maria, RS |
| SWSM04 | **3.1** | Swine | Santa Maria, RS |
| ATCC 27853 | **4.1** |  |  |
| CAPE01 | **5.1** | Canine | Pelotas, RS |
| CAPE02 | **5.2** | Canine | Pelotas, RS |
| BOIJ01 | **6** | Bovine | Ijuí, RS |
| BOIJ02 | **7.1** | Bovine | Julio de Castilhos, RS |
| CASM01 | **8.1** | Canine | Santa Maria, RS |
| CASM02 | **8.2** | Canine | Santa Maria, RS |
| CASM03 | **9.1** | Canine | Santa Maria, RS |
| CASM04 | **10.1** | Canine | Santa Maria, RS |
| CASM05 | **11.1** | Canine | Santa Maria, RS |
| CASM06 | **11.2** | Canine | Santa Maria, RS |
| BOJC01 | **12.1** | Bovine | Julio de Castilhos, RS |
| EQSM01 | **13** | Equine | Santa Maria, RS |
| EQSM02 | **14** | Equine | Santa Maria, RS |
| CASM07 | **16** | Canine | Santa Maria, RS |
| BOSV01 | **17.1** | Bovine | São Vicente do Sul |
| BOIJ03 | **18** | Bovine | Ijuí, RS |
| BOCA01 | **19** | Bovine | Cacequi, RS |
| CASM08 | **20.1** | Canine | Santa Maria, RS |
| CASM09 | **20.2** | Canine | Santa Maria, RS |
| BOPS01 | **21** | Bovine | Paraiso do Sul, RS |
| BOSJ01 | **22** | Bovine | São Jorge do Oeste, PR |
| BOCP01 | **23.1** | Bovine | Cunha Porã, SC |
| BOCP02 | **23.2** | Bovine | Cunha Porã, SC |
| CASM10 | **24.1** | Canine | Santa Maria, RS |
| BOSM01 | **25.1** | Bovine | Santa Maria, RS |
| ROSM01 | **26.1** | Rodent ^2^ | Santa Maria, RS |
| CASM11 | **27.1** | Canine | Santa Maria, RS |
| CASM12 | **28.1** | Canine | Santa Maria, RS |
| SWSM05 | **29.1** | Swine | Santa Maria, RS |
| BOSJ02 | **30** | Bovine | São Jorge do Oeste, PR |
| CASP01 | **31** | Canine | São Sepé, RS |
| CASM13 | **33.1** | Canine | Santa Maria, RS |

^1^ City and state name. Rio Grande do Sul (RS), Santa Catarina (SC) and Paraná (PR) are states from the Southern region of Brazil

^2^ Strain obtained from Chinchilla
