# Supplementary material for: Characterization of a bacteriophage with broad host range against strains of Pseudomonas aeruginosa isolated from domestic animals
Source: BMC Microbiol. 2019 Jun 17;19:134. doi: 10.1186/s12866-019-1481-z (PMC6580649; doi:10.1186/s12866-019-1481-z)
Supplement: Supplementary file 3 — Table S3. Genbank accession number of the 16S rRNA genes of strains that were not susceptible to phage BrSP1 (DOCX 12 kb) [file 12866_2019_1481_MOESM3_ESM.docx]

**Additional file 3: Table S3.** Genbank accession number of the 16S rRNA genes of *P. aeruginosa* strains that were not susceptible to phage BrSP1 and of strain Lfar01

| **Isolate** | **Genbank Accession number** |
| --- | --- |
| SWSM01 | MH094036 |
| SWSM02 | MH094037 |
| SWSM03 | MH094038 |
| SWSM04 | MH094039 |
| CASM01 | MH094040 |
| CASM02 | MH094041 |
| CASM04 | MH094042 |
| EQSM02 | MH094043 |
| EQSM03 | MH094044 |
| BOSV01 | MH094045 |
| CASM08 | MH094046 |
| CASM09 | MH094047 |
| BOPS01 | MH094048 |
| BOCP01 | MH094049 |
| BOCP02 | MH094050 |
| ROSM01 | MH094051 |
| CASM12 | MH094052 |
| SWSM05 | MH094053 |
| CASP01 | MH094036 |
| CASM13 | MH094038 |
| Lfar01 | MF590114.1 |
