# Supplementary material for: Characterization of a bacteriophage with broad host range against strains of Pseudomonas aeruginosa isolated from domestic animals
Source: BMC Microbiol. 2019 Jun 17;19:134. doi: 10.1186/s12866-019-1481-z (PMC6580649; doi:10.1186/s12866-019-1481-z)
Supplement: Supplementary file 4 — Table S4. ORFs identified in the genome of Pseudomonas phage BrSP1 (DOCX 41 kb) [file 12866_2019_1481_MOESM4_ESM.docx]

**Additional file 4: Table S4.** ORFs identified in the genome of Pseudomonas phage BrSP1

| **ORF*** | **Position** |  | **Strand** | **#AA** | **Phage Genus *Pbunavirus***** | **Structural Predictions***** | **e-value** | **Identity** |
| --- | --- | --- | --- | --- | --- | --- | --- | --- |
| **ORF-1*** | 4 | 411 | forward | **135** | Pseudomonas phage JG024 | hypothetical protein PJG24_078 | 7,00E-85 | 95% |
| **ORF-2** | 602 | 1.171 | forward | **189** | Pseudomonas phage LMA2 | hypothetical protein LMA2_gp76 | 9,00E-138 | 99% |
| **ORF-3** | 1.341 | 1.964 | reverse | **207** | Pseudomonas phage LMA2 | hypothetical protein LMA2_gp77 | 2,00E-141 | 98% |
| **ORF-4** | 2.154 | 2.855 | reverse | **233** | Pseudomonas phage phiKTN6 | hypothetical protein phiKTN6_077 | 2,00E-156 | 99% |
| **ORF-5** | 2.866 | 3.177 | reverse | **103** | Pseudomonas phage 14-1 | hypothetical protein PP141_gp79 | 8,00E-72 | 99% |
| **ORF-6** | 3.230 | 3.451 | reverse | **73** | Pseudomonas phage 14-1 | hypothetical protein PP141_gp80 | 3,00E-44 | 100% |
| **ORF-7** | 3.461 | 3.715 | reverse | **84** | Pseudomonas phage 14-1 | hypothetical protein PP141_gp81 | 1,00E-53 | 100% |
| **ORF-8** | 3.769 | 3.993 | reverse | **74** | Pseudomonas phage LBL3 | hypothetical protein LBL3_gp79 | 3,00E-47 | 100% |
| **ORF-9** | 4.059 | 4.385 | reverse | **108** | Pseudomonas phage LBL3 | hypothetical protein LBL3_gp80 | 5,00E-72 | 98% |
| **ORF-10** | 4.386 | 5.030 | reverse | **214** | Pseudomonas phage DL68 | hypothetical protein | 5,00E-155 | 99% |
| **ORF-11** | 5.062 | 5.271 | reverse | **69** | Pseudomonas phage Ab27 | hypothetical protein | 7,00E-40 | 94% |
| **ORF-12** | 5.268 | 5.483 | reverse | **71** | Pseudomonas phage 14-1 | hypothetical protein PP141_gp86 | 3,00E-43 | 99% |
| **ORF-13** | 5.480 | 5.677 | reverse | **65** | Pseudomonas phage LBL3 | hypothetical protein LBL3_gp85 | 8,00E-38 | 98% |
| **ORF-14** | 5.677 | 5.928 | reverse | **83** | Pseudomonas phage JG024 | hypothetical protein PJG24_092 | 3,00E-54 | 100% |
| **ORF-15*** | 6.014 | 6.208 | reverse | **64** | Pseudomonas phage DL68 | hypothetical protein | 1,00E-39 | 98% |
| **ORF-16** | 6.205 | 6.891 | reverse | **228** | Pseudomonas phage DL68 | capsid and scaffold protein | 2,00E-165 | 99% |
| **ORF-17** | 6.946 | 7.248 | reverse | **100** | Pseudomonas phage F8 | hypothetical protein ORF001 | 6,00E-63 | 98% |
| **ORF-18** | 7.263 | 7.409 | reverse | **48** | Pseudomonas phage F8 | hypothetical protein ORF002 | 2,00E-24 | 100% |
| **ORF-19** | 7.578 | 7.736 | reverse | **52** | Pseudomonas phage LBL3 | hypothetical protein LBL3_gp87 | 4,00E-32 | 100% |
| **ORF-20** | 7.907 | 9.289 | forward | **460** | Pseudomonas phage DL68 | terminase large subunit | 0.0 | 99% |
| **ORF-21** | 9.326 | 9.709 | reverse | **127** | Pseudomonas phage 14-1 | hypothetical protein PP141_gp04 | 2,00E-85 | 99% |
| **ORF-22** | 9.706 | 9.924 | reverse | **72** | Pseudomonas phage SN | hypothetical protein PPSN_gp06 | 2,00E-45 | 99% |
| **ORF-23** | 9.924 | 10.274 | reverse | **116** | Pseudomonas phage DL68 | hypothetical protein | 1,00E-78 | 100% |
| **ORF-24** | 10.319 | 10.693 | reverse | **124** | Pseudomonas phage KPP22 | hypothetical protein | 2,00E-83 | 100% |
| **ORF-25** | 10.720 | 11.499 | reverse | **259** | Pseudomonas phage Ab27 | hypothetical protein | 0.0 | 99% |
| **ORF-26** | 11.586 | 12.023 | reverse | **145** | Pseudomonas phage SN | hypothetical protein PPSN_gp10 | 5,00E-96 | 97% |
| **ORF-27** | 12.041 | 12.628 | reverse | **195** | Pseudomonas phage NP3 | hypothetical protein | 2,00E-143 | 98% |
| **ORF-28** | 12.638 | 12.733 | reverse | **31** | Pseudomonas phage SN | hypothetical protein PPSN_gp12 | 4,00E-11 | 94% |
| **ORF-29** | 12.730 | 13.662 | reverse | **310** | Pseudomonas phage Ab27 | hypothetical protein | 0.0 | 100% |
| **ORF-30** | 13.766 | 14.113 | reverse | **115** | Pseudomonas phage LBL3 | hypothetical protein LBL3_gp11 | 2,00E-76 | 99% |
| **ORF-31*** | 14.251 | 14.418 | forward | **55** | Pseudomonas phage vB_Pae_PS44 | hypothetical protein vB_Pae_PS44_00016 | 3,00E-30 | 96% |
| **ORF-32** | 14.362 | 14.907 | reverse | **181** | Pseudomonas phage JG024 | hypothetical protein PJG24_015 | 4,00E-121 | 93% |
| **ORF-33** | 14.879 | 15.202 | reverse | **107** | Pseudomonas phage LMA2 | hypothetical protein LMA2_gp15 | 3,00E-75 | 100% |
| **ORF-34** | 15.234 | 15.635 | reverse | **133** | Pseudomonas phage Ab27 | hypothetical protein | 3,00E-92 | 100% |
| **ORF-35** | 15.816 | 18.113 | forward | **765** | Pseudomonas phage SN | putative minor head protein | 0.0 | 99% |
| **ORF-36** | 18.113 | 18.949 | forward | **278** | Pseudomonas phage KPP12 | putative minor head protein | 0.0 | 100% |
| **ORF-37** | 18.968 | 19.174 | forward | **68** | Pseudomonas phage LBL3 | hypothetical protein LBL3_gp17 | 2,00E-41 | 100% |
| **ORF-38** | 19.171 | 19.311 | forward | **46** | Pseudomonas phage LMA2 | hypothetical protein LMA2_gp20 | 8,00E-23 | 98% |
| **ORF-39** | 19.825 | 21.258 | forward | **477** | Pseudomonas phage KPP12 | putative structural protein | 0.0 | 99% |
| **ORF-40** | 21.262 | 21.897 | forward | **211** | Pseudomonas phage SN | structural protein | 6,00E-148 | 100% |
| **ORF-41** | 21.907 | 23.055 | forward | **382** | Pseudomonas phage LMA2 | putative major structural protein | 0.0 | 99% |
| **ORF-42*** | 23.013 | 23.132 | forward | **39** | Pseudomonas phage vB_PaeM_E217 | nucleotide sequence* | 6,00E-54 | 100% |
| **ORF-43** | 23.157 | 23.594 | forward | **145** | Pseudomonas phage LBL3 | hypothetical protein LBL3_gp22 | 6,00E-104 | 100% |
| **ORF-44** | 23.609 | 24.076 | forward | **155** | Pseudomonas phage KPP12 | putative structural protein | 4,00E-110 | 100% |
| **ORF-45** | 24.100 | 24.471 | forward | **123** | Pseudomonas phage KPP12 | putative structural protein | 8,00E-85 | 100% |
| **ORF-46** | 24.479 | 25.030 | forward | **183** | Pseudomonas phage phiKTN6 | structural protein | 8,00E-134 | 100% |
| **ORF-47** | 25.027 | 25.608 | forward | **193** | Pseudomonas phage F8 | hypothetical protein ORF028 | 2,00E-137 | 100% |
| **ORF-48*** | 25.624 | 27.138 | forward | **504** | Pseudomonas phage SN | structural protein | 0.0 | 99% |
| **ORF-49** | 27.197 | 27.649 | forward | **150** | Pseudomonas phage LMA2 | putative structural protein | 2,00E-103 | 100% |
| **ORF-50** | 27.649 | 27.972 | forward | **107** | Pseudomonas phage LMA2 | putative structural protein | 2,00E-75 | 100% |
| **ORF-51** | 27.969 | 28.319 | forward | **116** | Pseudomonas phage LMA2 | putative structural protein | 2,00E-78 | 100% |
| **ORF-52** | 28.321 | 28.752 | forward | **143** | Pseudomonas phage KPP12 | putative structural protein | 3,00E-91 | 98% |
| **ORF-53** | 28.762 | 29.265 | forward | **167** | Pseudomonas phage SN | structural protein | 9,00E-115 | 99% |
| **ORF-54** | 29.400 | 29.804 | forward | **134** | Pseudomonas phage LMA2 | putative structural protein | 6,00E-94 | 100% |
| **ORF-55** | 29.698 | 29.859 | reverse | **53** | Pseudomonas phage vB_PaeM_E215 | nucleotide sequence* | 9,00E-78 | 100% |
| **ORF-56*** | 29.813 | 30.406 | forward | **197** | Pseudomonas phage S12-1 | Phage tail fiber protein | 8,00E-139 | 99% |
| **ORF-57** | 30.416 | 30.844 | forward | **142** | Pseudomonas phage LBL3 | hypothetical protein LBL3_gp35 | 4,00E-101 | 100% |
| **ORF-58** | 30.848 | 33.424 | forward | **858** | Pseudomonas phage vB_PaeM_E217 | transglycosylase | 0.0 | 99% |
| **ORF-59** | 33.424 | 34.287 | forward | **287** | Pseudomonas phage LMA2 | putative structural protein | 0.0 | 100% |
| **ORF-60** | 34.287 | 34.820 | forward | **177** | Pseudomonas phage DL68 | hypothetical protein | 5,00E-127 | 99% |
| **ORF-61** | 34.876 | 35.541 | forward | **221** | Pseudomonas phage SN | Chain A, Crystal structure of the cell puncturing protein gp41 | 1,00E-159 | 99% |
| **ORF-62** | 35.605 | 36.858 | forward | **417** | Pseudomonas phage NH-4 | putative baseplate protein | 0.0 | 99% |
| **ORF-63** | 36.855 | 38.369 | forward | **504** | Pseudomonas phage SN | structural protein | 0.0 | 97% |
| **ORF-64*** | 38,389 |  | forward | **957** | Pseudomonas phage NP3 | tail fibers protein | 0.0 | 98% |
| **ORF-65** | 41.264 | 41.692 | forward | **142** | Pseudomonas phage JG024 | putative tail fiber component | 2,00E-94 | 96% |
| **ORF-66** | 41.692 | 42.354 | forward | **220** | Pseudomonas phage KPP12 | putative endolysin | 7,00E-162 | 99% |
| **ORF-67** | 42.379 | 42.630 | reverse | **83** | Pseudomonas phage 14-1 | hypothetical protein PP141_gp48 | 2,00E-50 | 100% |
| **ORF-68** | 42.910 | 43.821 | reverse | **303** | Pseudomonas phage LMA2 | putative DNA ligase | 0.0 | 98% |
| **ORF-69** | 43.876 | 44.430 | reverse | **184** | Pseudomonas phage vB_PaeM_LS1 | DNA-binding protein | 1,00E-131 | 99% |
| **ORF-70** | 44.427 | 45.032 | reverse | **201** | Pseudomonas phage F8 | hypothetical protein ORF050 | 3,00E-138 | 99% |
| **ORF-71** | 45.087 | 45.986 | reverse | **299** | Pseudomonas phage DL68 | hypothetical protein | 0.0 | 100% |
| **ORF-72** | 46.075 | 46.770 | reverse | **231** | Pseudomonas phage LMA2 | hypothetical protein LMA2_gp52 | 3,00E-141 | 99% |
| **ORF-73** | 46.865 | 48.424 | reverse | **519** | Pseudomonas phage vB_Pae436M-8 | DNA helicase | 0.0 | 99% |
| **ORF-74** | 48.421 | 48.831 | reverse | **136** | Pseudomonas phage LBL3 | hypothetical protein LBL3_gp52 | 5,00E-95 | 100% |
| **ORF-75*** | 48.824 | 52.042 | reverse | **1072** | Pseudomonas phage vB_Pae_PS44 | DNA polymerase III subunit alpha | 0.0 | 99% |
| **ORF-76** | 51.931 | 52.485 | reverse | **184** | Pseudomonas phage LMA2 | putative DNA polymerase III, epsilon subunit | 1,00E-134 | 99% |
| **ORF-77** | 52.562 | 53.620 | reverse | **352** | Pseudomonas phage KPP12 | putative polynucleotide kinase | 0.0 | 95% |
| **ORF-78** | 53.623 | 53.814 | reverse | **63** | Pseudomonas phage KPP12 | hypothetical protein | 7,00E-36 | 100% |
| **ORF-79** | 53.816 | 54.733 | reverse | **305** | Pseudomonas phage KPP12 | putative thymidylate synthase | 0.0 | 99% |
| **ORF-80** | 54.733 | 54.939 | reverse | **68** | Pseudomonas phage F8 | hypothetical protein ORF060 | 3,00E-42 | 100% |
| **ORF-81** | 54.951 | 55.217 | reverse | **88** | Pseudomonas phage F8 | hypothetical protein ORF061 | 6,00E-56 | 97% |
| **ORF-82** | 55.217 | 55.435 | reverse | **72** | Pseudomonas phage DL52 | tail assembly protein | 2,00E-46 | 100% |
| **ORF-83** | 55.419 | 55.637 | reverse | **72** | Pseudomonas phage DL52 | hypothetical protein | 3,00E-43 | 100% |
| **ORF-84** | 55.637 | 55.867 | reverse | **76** | Pseudomonas phage PB1 | hypothetical protein PB1_gp67 | 3,00E-49 | 99% |
| **ORF-85** | 55.957 | 56.958 | reverse | **333** | Pseudomonas phage DL60 | hypothetical protein | 0.0 | 99% |
| **ORF-86** | 57.063 | 57.953 | reverse | **296** | Pseudomonas phage KPP12 | putative structural protein | 0.0 | 98% |
| **ORF-87** | 58.114 | 59.301 | reverse | **395** | Pseudomonas phage DL52 | DNA helicase | 0.0 | 99% |
| **ORF-88** | 59.288 | 59.710 | reverse | **140** | Pseudomonas phage NP3 | hypothetical protein | 9,00E-98 | 99% |
| **ORF-89** | 59.879 | 60.664 | forward | **261** | Pseudomonas phage 14-1 | hypothetical protein PP141_gp70 | 0.0 | 99% |
| **ORF-90** | 60.675 | 62.204 | forward | **509** | Pseudomonas phage E215 | hypothetical protein vBPaeME215_00050 | 0.0 | 76% |
| **ORF-91** | 62.228 | 62.677 | forward | **149** | Pseudomonas phage 14-1 | hypothetical protein PP141_gp72 | 3,00E-106 | 100% |
| **ORF-92** | 62.674 | 63.750 | forward | **358** | Pseudomonas phage vB_Pae436M-8 | DNA primase | 0.0 | 99% |
| **ORF-93** | 63.756 | 63.941 | forward | **61** | Pseudomonas phage LMA2 | hypothetical protein LMA2_gp73 | 8,00E-34 | 100% |
| **ORF-94** | 64.089 | 65.819 | forward | **576** | Pseudomonas phage DL60 | DNA primase | 0.0 | 98% |

* Eight putative genes initiated with alternative initiation codons (ORFs 1, 15, 31, 42, 48, 56, 64 and 75);

** The Pseudomonas phage with the highest similarity are indicated;

*** Structural prediction for the putative proteins with highest similarity;
