# Supplementary material for: Characterization of a bacteriophage with broad host range against strains of Pseudomonas aeruginosa isolated from domestic animals
Source: BMC Microbiol. 2019 Jun 17;19:134. doi: 10.1186/s12866-019-1481-z (PMC6580649; doi:10.1186/s12866-019-1481-z)
Supplement: Supplementary file 5 — Table S5. Codon usage in phage BrSP1 and in P. aeruginosa (PAO) (DOCX 18 kb) [file 12866_2019_1481_MOESM5_ESM.docx]

**Additional file 5: Table S5.** Codon usage in phage BrSP1 and *in P. aeruginosa* (PAO) ***

|  | **RSCU** |  |  |  |  | **RSCU** |  |
| --- | --- | --- | --- | --- | --- | --- | --- |
|  | **BrSP1** | **PAO** |  |  |  | **BrSP1** | **PAO** |
| alanine |  |  |  |  |  |  |  |
| GCA | 0,53 | 0,17 |  |  | lysine |  |  |
| GCC | 1,71 | 2,33 |  |  | AAA | 0,73 | 0,25 |
| GCG | 1,09 | 1,34 |  |  | AAG | 1,27 | 1,75 |
| GCT | 0,67 | 0,17 |  |  |  |  |  |
|  |  |  |  |  | leucine |  |  |
| cysteine |  |  |  |  | CTA | 0,24 | 0,07 |
| TGC | 1,52 | 1,80 |  |  | CTC | 1,33 | 1,34 |
| TGT | 0,48 | 0,20 |  |  | CTG | 2,77 | 4,01 |
|  | 2,00 | 2,00 |  |  | CTT | 0,71 | 0,15 |
|  |  |  |  |  | TTA | 0,08 | 0,01 |
| aspartic acid |  |  |  |  | TTG | 0,87 | 0,42 |
| GAC | 1,12 | 1,61 |  |  |  |  |  |
| GAT | 0,88 | 0,39 |  |  | asparagine | |  |
|  |  |  |  |  | AAC | 1,37 | 1,72 |
| glutamic acid | |  |  |  | AAT | 0,63 | 0,28 |
| GAA | 1,09 | 0,77 |  |  |  |  |  |
| GAG | 0,91 | 1,23 |  |  | proline |  |  |
|  |  |  |  |  | CCA | 0,58 | 0,17 |
| phenylalanine | |  |  |  | CCC | 0,57 | 1,03 |
| TTC | 1,70 | 1,90 |  |  | CCG | 2,06 | 2,63 |
| TTT | 0,30 | 0,10 |  |  | CCT | 0,80 | 0,17 |
|  |  |  |  |  |  |  |  |
| glycine |  |  |  |  | glutamine |  |  |
| GGA | 0,67 | 0,20 |  |  | CAA | 0,67 | 0,29 |
| GGC | 2,13 | 2,94 |  |  | CAG | 1,33 | 1,71 |
| GGG | 0,52 | 0,47 |  |  |  |  |  |
| GGT | 0,68 | 0,39 |  |  | arginine |  |  |
|  |  |  |  |  | AGA | 0,37 | 0,04 |
| histidine |  |  |  |  | AGG | 0,43 | 0,16 |
| CAC | 1,29 | 1,42 |  |  | CGA | 0,71 | 0,19 |
| CAT | 0,71 | 0,58 |  |  | CGC | 2,58 | 3,88 |
|  |  |  |  |  | CGG | 1,16 | 1,11 |
|  |  |  |  |  | CGT | 0,75 | 0,62 |
| isoleucine |  |  |  |  |  |  |  |
| ATA | 0,24 | 0,07 |  |  | threonine |  |  |
| ATC | 2,07 | 2,73 |  |  | ACA | 0,30 | 0,08 |
| ATT | 0,69 | 0,21 |  |  | ACC | 1,86 | 3,15 |
|  |  |  |  |  | ACG | 0,84 | 0,61 |
| serine |  |  |  |  | ACT | 1,00 | 0,16 |
| AGC | 1,36 | 2,83 |  |  |  |  |  |
| AGT | 0,49 | 0,29 |  |  | valine |  |  |
| TCA | 0,33 | 0,06 |  |  | GTA | 0,49 | 0,23 |
| TCC | 1,56 | 1,31 |  |  | GTC | 1,55 | 1,67 |
| TCG | 1,43 | 1,42 |  |  | GTG | 1,12 | 1,94 |
| TCT | 0,83 | 0,09 |  |  | GTT | 0,84 | 0,16 |
|  |  |  |  |  |  |  |  |
| tyrosine |  |  |  |  | Stop codon | |  |
| TAC | 0,98 | 1,58 |  |  | TAA | 1,24 | 0,28 |
| TAT | 1,02 | 0,42 |  |  | TAG | 0,35 | 0,34 |
|  |  |  |  |  | TGA | 1,40 | 2,37 |

* Data from Grocock RJ, Sharp PM, 2002
